# Navigating the Neuroimmunomodulation Frontier: Pioneering Approaches and Promising Horizons—A Comprehensive Review

**DOI:** 10.3390/ijms25179695

**Published:** 2024-09-07

**Authors:** Antea Krsek, Leona Ostojic, Dorotea Zivalj, Lara Baticic

**Affiliations:** 1 Faculty of Medicine, University of Rijeka, 51000 Rijeka, Croatia; tea.krsek@gmail.com (A.K.); lostojic@student.uniri.hr (L.O.); dorotea.zivalj@gmail.com (D.Z.); 2 Department of Medical Chemistry, Biochemistry and Clinical Chemistry, Faculty of Medicine, University of Rijeka, 51000 Rijeka, Croatia

**Keywords:** cytokines, immune system, inflammatory illnesses, microglial cells, mood disorders, neuroimmunomodulation, neurofeedback, vagus nerve stimulation

## Abstract

The research in neuroimmunomodulation aims to shed light on the complex relationships that exist between the immune and neurological systems and how they affect the human body. This multidisciplinary field focuses on the way immune responses are influenced by brain activity and how neural function is impacted by immunological signaling. This provides important insights into a range of medical disorders. Targeting both brain and immunological pathways, neuroimmunomodulatory approaches are used in clinical pain management to address chronic pain. Pharmacological therapies aim to modulate neuroimmune interactions and reduce inflammation. Furthermore, bioelectronic techniques like vagus nerve stimulation offer non-invasive control of these systems, while neuromodulation techniques like transcranial magnetic stimulation modify immunological and neuronal responses to reduce pain. Within the context of aging, neuroimmunomodulation analyzes the ways in which immunological and neurological alterations brought on by aging contribute to cognitive decline and neurodegenerative illnesses. Restoring neuroimmune homeostasis through strategies shows promise in reducing age-related cognitive decline. Research into mood disorders focuses on how immunological dysregulation relates to illnesses including anxiety and depression. Immune system fluctuations are increasingly recognized for their impact on brain function, leading to novel treatments that target these interactions. This review emphasizes how interdisciplinary cooperation and continuous research are necessary to better understand the complex relationship between the neurological and immune systems.

## 1. Introduction

At the vanguard of quickly developing fields in the biological sciences, neuroimmunology bridges the critical divide between the immune and neurological systems [1]. Understanding how closely linked these two seemingly different systems are is at the core of neuroimmunomodulation. The central nervous system and immune system have an active communication relationship that goes beyond a simple link. This complex web of communication affects many aspects of our lives, including illness, health, and how we react to different environmental and psychological stimuli. A crucial component of the two systems’ reciprocal communication is the nervous system’s regulation of immunological responses [2]. The benefits of the capability of the nervous system to regulate immunity are examined, as are the possible advantages resulting from the brain’s special functions, such as its ability to integrate physiological processes, make predictions, and react quickly. The communication channels between the brain and peripheral immune system are examined, encompassing the endocrine, sympathetic, parasympathetic, sensory, and meningeal lymphatic systems. Furthermore, the processing and regulation of immune information in the brain regions are investigated, providing a partial map to guide the conceptual framework for the generation of hypotheses and the study of these intricate interactions [1].

The immune system’s receptors for neurotransmitters such as acetylcholine and norepinephrine, as well as the sympathetic nervous system’s (SNS’s) fibers’ attachment to lymph nodes, enable these controls [3]. As previously indicated, situated at the nexus of immunology and neurology, neuroimmunomodulation provides an engaging investigation of the complex conversation between our immunological and neurological systems. This dynamic interaction has important ramifications for understanding the dynamics of both health and illness [4]. The relationships between the neurological system and the immune system have mostly been studied in the context of illnesses. However, recent studies are shedding light on the ways in which certain soluble effectors, known as cytokines, which are made by immune cells, can influence host behavior even when there is not an active infection. Every mechanism is most likely developed to maximize an organism’s ability to respond to external challenges, hence raising its odds of surviving [5].

There are remarkable similarities between the neurological and immunological systems, which serve as links between the internal systems and exterior surroundings. Specialized sensors designed to detect environmental and internal inputs are present in every system. Immune system lymphocytes, for example, display TCRs, or T-cell receptors, and B-cell receptors, and different immune cells use pattern-recognition receptors (PRRs) to identify pathogens [6]. Similarly, sensory neurons in the nervous system, including nociceptors, express a variety of ligand-gated or voltage-gated channels, allowing them to register information about noxious stimuli [7]. Additionally, immune cells feature receptors responsive to neurotransmitters and neuropeptides, exerting influence over inflammation and immunosuppression processes [8,9,10,11,12]. Functional pattern-recognition receptors, including Toll-like receptor 3 (TLR3), TLR4, TLR7, and TLR9, as well as cytokine receptors, are expressed by sensory neurons. This allows them to recognize pathogens and danger signals, and it makes nociceptors more sensitive to pain and itching. These receptors’ reciprocal expression raises the possibility of communication between the neurological and immunological systems [5,13,14]. The organism shows an extensive distribution of both systems. While neurons use the expansion of lengthy processes, immune cells use the circulation of blood to monitor tissue. This enables fast response propagation upon danger signal detection and ongoing tissue surveillance. Additionally, both systems exhibit an elevated level of flexibility that allows them to adapt to different situations. Because of these commonalities, evolution may have an advantage in identifying and reacting to environmental stresses [5].

Every system employs distinct strategies to ensure the host’s survival. By combining information from the outside and within, the nervous system controls behavior. When faced with drugs or circumstances that are thought to be hazardous, avoidance behavior is triggered. The immune system defends the organism against harmful infection, damage, or stress by using processes of resistance or tolerance. New research reveals that the immune system can control behavior in addition to its usual functions, underscoring its neglected function as a neuromodulator [5,15,16,17].

### Tracing the Roots of Neuroimmunomodulation

Over the years of its fascinating history, neuroimmunomodulation has undergone significant advancements that have shed light on its major consequences for human well-being and illness. The initial focus was on brain-to-immune communication channels, partly because of the keen interest of neuroendocrinologists and the progress made in understanding the structure of chemicals and binding mechanisms of neuroendocrine hormones. Over time, cells of endhothelial cells, glia, and neurons have developed complex functional and structural relationships, and neurobiologists have come to understand the significance of cytokines locally generated by brain cells in these interactions. It was long thought that immune chemicals in bloodstreams functioning primarily on the brain were what caused the brain’s connection to the nervous system.

Early 20th-century research on psychological effects on immunity at the Institute Pasteur in the French capital led to revolutionary discoveries that eventually shaped the field of neuroimmunomodulation [2]. However, it was not until the 1970s that observational evidence from Ader and Cohen of the complex interaction connecting the immune system and the central nervous system restored conditioned immunosuppression to the mainstream [18]. This significant finding has rekindled scientific interest in understanding the neuroimmunomodulatory pathways [19]. Hans Selye is believed to be the first scientist to identify ‘stress’ as underpinning the nonspecific signs and symptoms of illness and the founder of the “stress theory”. He distinguished acute stress from the total response to chronically applied stressors, terming the latter condition ‘general adaptation syndrome’, which is also known in the literature as Selye’s Syndrome [20].

Neuroimmunoendocrinology, neuroimmunomodulation, and psychoneuroimmunology are names given to the diverse fields of study on neural-immune interactions, depending on the dominant scientific discipline [4]. The intricate connections between the brain system, endocrine components, and immunological system were encapsulated in these designations. The discipline of neuroimmunomodulation developed as a result of the finding of pathways via which the neurological and immune systems communicate. Neuroendocrine peptide hormones had a major impact on this communication, which in turn affected immune responses. Neurotransmitter release from both main and secondary lymphoid organ nerve endings, including norepinephrine and adrenaline, has also been identified as an essential system in long-range communication routes. A crucial moment in research was the finding that immune cells could create and discharge their neuroendocrine hormones and neuromodulators [2]. When the focus switched from far-reaching to short-range pathways for interaction within the neurological and immune systems, highlighting the importance of local interactions, an essential turning point in the area of neuroimmunomodulation was attained.

The growing amount of evidence linking immunological senescence to the development of late-stage neurodegenerative illnesses has led to a major expansion in the field of neuroimmunology over the last two decades. Moreover, the discovery that adult neurons lack the complement component C1q but postnatal neurons have it in their synapses emphasizes the importance of the immune system in brain development [21]. Recent findings suggest that an individual’s susceptibility to neuroinflammatory disorders may be influenced by their gut microbiota and bacteria such as the resurgent Zika and Ebola viruses.

Clinical investigations have demonstrated the need for more specialized approaches than overall immunosuppression in the treatment of neuroimmune disorders. In order to rectify genetic mutations, methods such as gene editing, stem cell treatment, tolerance induction, and cell loss (e.g., B cells in MS) have been gaining popularity [22]. By revealing the course of the disease and the function of inflammation, modern imaging techniques like optical coherence tomography, single photon emission computed tomography (CT), PET (Positron emission tomography), ligands, and high-resolution magnetic resonance imaging (MRI) are improving the study of neuroinflammatory diseases in patients [23,24]. In vivo optical imaging using GFP-labeled T lymphocytes, glia, and transplanted pluripotent stem cells induced by humans has revolutionized our understanding of the interaction among the immune and neurological systems in animal models. Genetic modification has been shown to be a vital technique for examining gene functionality in both natural development and disease. It has primarily been studied in vitro and in animal investigations. This includes the development of novel genome editing tools and gene-targeting techniques, of which CRISPR/Cas9 is an outstanding example [25]. This gene-editing approach has made it possible to genetically modify human iPSCs, which can now be used as tools for targeting viral infections and as models for ALS, while its application to human illnesses is still being studied [26].

**Figure 1:**
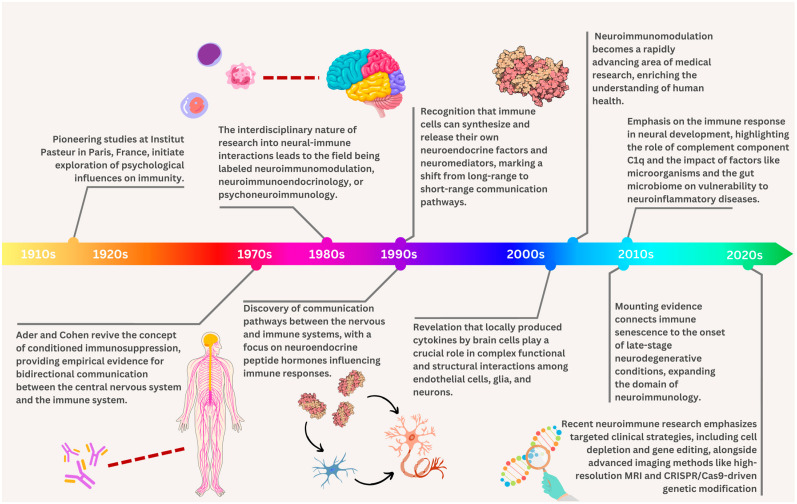
Timeline of neuroimmunomodulation research, tracing the evolution of neuroimmunomodulation from early 20th-century investigations at Institute Pasteur to contemporary advances in clinical trials and genetic modification.

The historical study of neuroimmunomodulation presents an intriguing journey marked by significant discoveries that have affected our understanding of the intricate relationship that exists between the immune and neurological systems. This journey demonstrates the changing landscape of a field of study that continuously produces new insights into the mutual relationship between the nervous and immune systems. It begins with the early investigations of immune cell neurotransmitter receptors and ends with the more recent deciphering of complex molecular mechanisms. Figure 1 shows the most important facts and break-through findings in the field of neuroimmunomodulation.

## 2. Techniques in Neuroimmunomodulation

The advent of novel technology has fueled recent advancements in neuroimmunology by providing a more human-centered understanding of immunological systems [27]. These developments are particularly helpful in the study of uncommon neuroimmunology conditions such as type 1 narcolepsy, Rasmussen encephalitis, and Susac syndrome. Under such circumstances, these technologies facilitate the integration of data from investigations involving both humans and animals, validating pathomechanistic characteristics and advancing the development of diagnostic and treatment approaches [28]. In the field of multiple sclerosis (MS), a thorough understanding of neuroimmunology has been crucial for developing novel treatments, and cutting-edge research methods offer vital diagnostic and prognostic resources [29]. It is projected that the combination of various technology techniques will advance our knowledge of neuroimmunological illnesses and improve patient outcomes [27].

Rapid developments in the field of neuroimmunomodulation are bringing new approaches to modify neuronal activity and treat nervous system problems. Optogenetics is one of these methods; it combines genetic manipulation and optics to control the activity of particular cells [30]. Furthermore, cutting-edge approaches like transcranial magnetic stimulation and deep brain stimulation are being researched for potential application in the management of neurological and mental health conditions [31]. Even though these approaches have shown encouraging results, there are still obstacles to be addressed, such as improving their efficacy and understanding the mechanisms of action [32]. To further advance the subject of neuroimmunomodulation, researchers are also looking into the application of water-dispersible carbon nanotubes and computational biology techniques [33].

### 2.1. Pharmacology

At the intersection of the fields of neuroscience, immunological research, and drugs, neuroimmune pharmacology is a young field that seeks to advance our understanding of disease mechanisms through translational research. The immunological elements of the central nervous system (CNS) are the focus of this discipline. The CNS is greatly influenced by both internal and external stimuli, including drugs of abuse, pathogenic microorganisms, and beneficial medicines [34].

In the treatment of neuroimmunological disorders, particularly in neuromuscular diseases, immunosuppressive medications play a vital role [35]. These therapeutic agents, which encompass corticosteroids, plasma exchange, and intravenous immunoglobulin IgG, have significantly influenced the treatment landscape for these conditions [36]. Notwithstanding, the intricacy of determining suitable treatments is noteworthy, considering the lack of conclusive protocols and the need to differentiate between basic neurological syndromes and those linked to systemic illnesses [37]. Despite the challenges involved, the ongoing progress in immunomodulatory drugs presents encouraging prospects for the management of autoimmune neurological diseases [38].

Immunomodulatory drugs, including methotrexate, azathioprine, cyclophosphamide, rituximab, glucocorticoids, mycophenolate, and intravenous immunoglobulins, are specifically intended to target and modify immune responses in neuroinflammatory conditions. These pharmaceuticals aim to restore immune balance without inducing widespread immunosuppression, necessitating careful management due to the potential for severe adverse effects [39]. The interplay among the immune, endocrine, and nervous systems is intricate, and disruptions within this network can lead to disease [40]. Consequently, the development and utilization of immunomodulatory medications represent a targeted approach to neuroimmunomodulation, holding the potential to enhance the treatment of neuroinflammatory conditions.

Distinguishing between immunosuppressive and immunomodulatory drugs holds pivotal significance when tailoring treatment approaches for neuroimmunology disorders [41]. Achieving the right equilibrium is vital to counteract pathological immune responses while safeguarding the body’s capacity to defend against infections and uphold overall health [35]. Pharmacological interventions are essential in the field of neuroimmunomodulation because they provide medical practitioners with exact control over immunity, hence reducing the detrimental effects of neuroinflammatory illnesses on the neurological system [38]. It is essential to acknowledge, however, that individuals undergoing these therapies face the risk of neurologic infections, presenting challenges in diagnosis and treatment [42].

### 2.2. Stimulation of the Vagus Nerve

**Figure 2:**
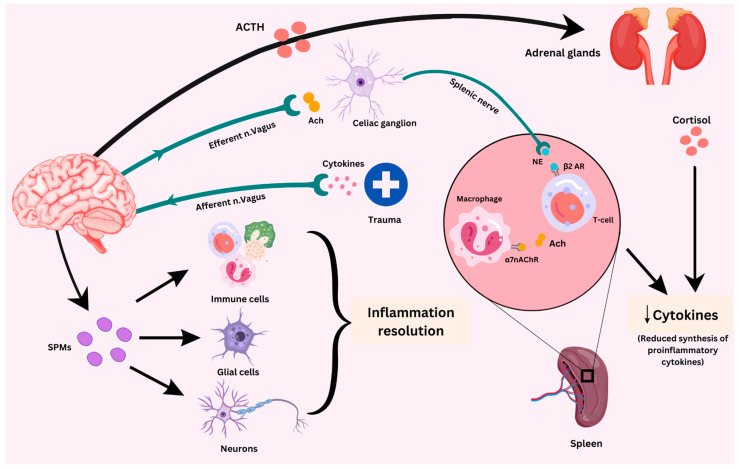
The network underpinning the vagus nerve stimulation’s anti-inflammatory actions in response to trauma. Specialized pro-resolving mediators (SPMs), which have anti-inflammatory properties, are released by the central nervous system as a result of afferent impulses from the vagus nerve (Afferent n.Vagus). These SPMs coordinate a coordinated response that includes neurons, glial cells, and immune cells, ultimately leading to the clearance of inflammation. The splenic nerve and spleen are impacted by the modulation of celiac ganglion activity by the efferent arm of the vagus nerve (Efferent n.Vagus). When this route is activated, acetylcholine is released, which affects the immune cells in the spleen and reduces the synthesis of cytokines.

The vagus nerve is the longest nerve in its subsection and the eleventh cranial nerve overall. It is necessary to provide two-way communication between the internal structures and the brain. Its origins can be traced back to its role in preserving autonomic homeostasis. Ten to twenty percent of the nerve is made up of myelinated efferent fibers, with the remaining eighty to ninety percent being unmyelinated sensory afferent fibers. By promoting interaction with the central, cardiopulmonary, as well as intestinal nervous systems, the vagal afferent and efferent nerves of the parasympathetic autonomic nerve system have two distinct roles that impact immunomodulation, enteroendocrine functions, and mental and emotional processes, which are summarized in Figure 2 [43].

A contemporary method of stimulating the vagus nerve using electrical signals is called vagus nerve stimulation (VNS) which can be carried out in an invasive or non-invasive manner. An electrode cuff is placed into the left cervical vagus nerve during conventional VNS. Additionally, visible in the subcutaneous region of the left anterior chest is an embedded electrical generator. The FDA has approved the use of traditional VNS as an adjuvant therapy for depression and drug-resistant epilepsy [44]. On the other hand, extracorporeally delivering electrical stimulation using transcutaneous or percutaneous techniques targets the cervical or auricular vagus nerve segments in non-invasive vagus nerve stimulation (VNS). This approach has been studied through research on treating a range of illnesses in both people and animals [45].

Though the exact mode of action of VNS is still unknown, theories indicate that it acts by stimulating vagal afferents and efferents, which stretch to internal organs and up to the brain. Vagal afferents influence brain activity, neurotransmitters, and endocrine functions connected to the hypothalamic–pituitary–adrenal axis by signaling the stimulation of brainstem nuclei and relayed cortical projections. Vagal efferent fibers are widely distributed throughout internal organs and function as a communication channel between the nervous system and the immune system, primarily through the cholinergic anti-inflammatory axis. The therapeutic advantages of vagus nerve stimulation are assumed to be based on alterations of neuronal circuits, neuroendocrine processes, and neuroimmune reactions; they are mediated by cholinergic anti-inflammatory pathway-mediated neuroimmunomodulation [43].

The vagal efferent route, a crucial part of the cholinergic anti-inflammatory process, regulates inflammatory responses. When this pathway is activated by the brain, the vagus nerve terminals produce acetylcholine (ACh). ACh, in turn, binds to α7 nicotinic acetylcholine receptors (α7nAChRs), which are present in a range of cell types, including liver Kupffer cells and splenic macrophages, to prevent the generation of inflammatory cytokines like TNF-α [46,47]. ACh also has the power to stop CD4+ T-cell maturation [48]. This method has been suggested as a potential therapeutic option for neurological diseases [49]. It has been demonstrated that it plays a part in how infectious diseases and inflammatory disorders regulate inflammation [50].

In particular, the gastrointestinal (GI) tract is where inflammation is mostly controlled by the cholinergic anti-inflammatory system. This route, which is triggered by the vagus nerve, interacts with enteric neurons and causes splenic macrophages to produce less TNF-α [51]. Oral NaHCO_3_ consumption is another method of stimulation; this initiates a splenic anti-inflammatory response that is conveyed to the spleen via a new mesothelial cell function resembling a neuron [52]. In this context, splenic macrophages with α7 nicotinic ACh receptors are stimulated by cholinergic T cells in the spleen, which serve as the initial source of acetylcholine [53]. Furthermore, by encouraging macrophages to perform tissue repair, sensory neurons—especially those that express the neuropeptide TAFA4—contribute to the anti-inflammatory reaction [54]. Research has particularly revealed that VNS significantly reduces TNF generation in wild-type mice, and that this reduction is lessened in animals with α7 receptor loss, emphasizing the critical roles that ACh and α7nAChR play in VNS’s anti-inflammatory pathway [55]. In addition, VNS has shown promise as an anti-inflammatory treatment when used in conjunction with early perinatal hypoxic brain damage [56].

It has been demonstrated that VNS dramatically lowers the number of activated macrophages and microglia in addition to the levels of cytokines associated with inflammation in the brain from mice with lipopolysaccharide-induced inflammation [57]. 12/15-lipoxygenase participates in this anti-inflammatory reaction, which is mediated by α7nAChR. In a rat model of endotoxemia, a study by Caravaca similarly found that VNS stabilized hemodynamic responses and decreased the plasma levels of multiple cytokines. Furthermore, VNS therapy caused a change in lipid mediators from pro-inflammatory to pro-resolving when given to animals with peritonitis [58]. All of these results point to the therapeutic value of VNS as a means of reducing neuroinflammation and controlling inflammatory reactions.

### 2.3. Neurofeedback

As a form of treatment, neurofeedback—also known as EEG biofeedback or neurotherapy—monitors brainwave activity in real time and provides feedback to patients to assist them in learning how to self-regulate their brain function [59]. The fundamental idea behind neurofeedback is that people can improve their behavioral and mental capacities by consciously controlling their brain activity. In this model, electroencephalography is the standard technique used to measure cerebral electrical activity. Neurofeedback techniques use attached scalp sensors to collect and capture patterns of brainwave activity. People are then exposed to this recorded data via visual or auditory stimuli, which frequently take the shape of images, sounds, or interactive video game formats [60]. Interestingly, neurofeedback shows promise in improving attentional deficiencies, especially when applied to treat ADHD (attention deficit hyperactivity disorder). Additionally, its benefits include lowering anxiety and stress, improving memory, learning, and abilities to solve problems, and improving mood and emotional control. In the medical landscape, this approach also takes on a complementary role and may be beneficial for neurological diseases like epilepsy and migraines. Furthermore, neurofeedback is a particularly helpful instrument for enhancing mental clarity and efficiency, especially in the domains of high-performance sports and high-level employment [61].

One potential treatment option for diseases like MDD that are mediated by neuroinflammation is real-time functional magnetic resonance imaging neurofeedback, or rtfMRI-nf [62]. Acknowledging the importance of neuroplasticity—the continual remodeling of brain structure and function throughout life—is essential to comprehend the implications of rtfMRI-nf. Reduced hippocampus volume and changed neuroplasticity-related gene expression have been found in MDD imaging investigations, highlighting the need to treat these brain disorders [63,64]. An essential component of neuroplasticity, synaptic plasticity, is greatly influenced by the immune system [65,66]. Pro-inflammatory cytokines in particular affect alterations in the kynurenine pathway’s metabolism (KP). This mechanism transforms tryptophan (TRP) into neuroactive compounds like quinolinic acid (QA) and kynurenic acid (KynA). QA, renowned because of its neurotoxic impacts, and KynA, believed to be neuroprotective, represent two competing forces in the fragile equilibrium among glutamatergic transmission with synaptic plasticity [67,68]. In this complex interaction, rtfMRI-nf exhibits potential as a regulator of the immune system’s impact on neuroplasticity. rtfMRI-nf may help to rebalance the KP by giving people access to real-time information about their brain activity, which would promote the creation of neuroprotective KynA over neurotoxic QA. This modification may provide a biological channel via which rtfMRI-nf ameliorates depressive symptoms by influencing glutamatergic transmission and synaptic plasticity [68,69]. Although there is growing evidence that neurofeedback is successful in certain fields, it is important to recognize that the discipline is dynamic and that more research is required to fully understand how it works and its usefulness in a range of uses [59].

### 2.4. Transcranial Magnetic Stimulation

Focused magnetic fields akin to those used in magnetic resonance imaging (MRI) are utilized in transcranial magnetic stimulation (TMS), an innovative, non-invasive neurostimulation technique. Using a state-of-the-art method, precise magnetic pulses are produced and carefully aimed to stimulate particular brain regions. Magnetic pulses generate a slight electrical current, facilitating the opening of neural connections in that specific area [70,71]. This method has shown intriguing links with neuroimmunomodulation, particularly in therapeutic contexts. TMS may affect the immune system by influencing the central nervous system, according to research [72]. Within the CNS, TMS can affect glial cells, with a particular emphasis on the prominent modulation of astrocytes. Astrocytes, crucial for metabolic support and synapse formation, show varied responses to TMS, influencing factors like glial fibrillary acidic protein (GFAP) expression and astrocytic activation. TMS-induced changes in synapse numbers and morphology implicate astrocytes as key mediators, impacting synaptic structure and efficacy. Astrocytes likely modulate glutamate uptake and release in response to TMS, contributing to the observed therapeutic effects. TMS may also impact oligodendrocytes, the cells responsible for myelinating axons. While the direct effect remains unexplored, TMS could potentially influence oligodendrogenesis by stimulating oligodendrocyte precursor cells (OPCs) and increasing brain-derived neurotrophic factor (BDNF), enhancing axonal ensheathing and myelin development [73].

TMS was first developed as a technique for neurological research, mainly for the study of brain mapping and motor skills. Its uses have broadened over time to encompass therapeutic and diagnostic procedures like cranial and spinal neurosurgery, rehabilitation for peripheral and central motor dysfunctions, and hemisphere dominance research. In these situations, single-pulse and repeating TMS (rTMS) have proven to be useful methods [74]. Multiple transcranial magnetic stimulation has been shown in preclinical studies to be beneficial in reducing depression-like symptoms, indicating that this treatment modality may be a good fit for major depressive disorder [75]. The application of this non-invasive therapeutic approach in treating neuropsychiatric disorders in children and adolescents has also been investigated [76]. Targeting the medial prefrontal cortex (mPFC) in studies on the brain, rTMS has demonstrated signs of improving symptoms in major depressive disorder, post-traumatic stress disorder, and obsessive–compulsive disorder [77]. In addition, it has been discovered that rTMS influences immunological markers in MDD patients, indicating its immunomodulatory properties [78]. These results demonstrate the promise of rTMS in the management of mental illnesses, such as MDD, and the necessity of more study to fully comprehend its mechanisms and maximize its application. Additionally, TMS has exhibited promise in both diagnosing and managing dementia, particularly in primary degenerative diseases like Alzheimer’s and vascular dementia [79]. However, its application in secondary degenerative and inflammatory diseases is less explored. TMS, with its measures of cortical function and plasticity—such as short-latency afferent inhibition, short-interval intracortical inhibition, and the cortical silent period—could potentially offer valuable insights in these less investigated cases. Additionally, TMS has been used to find early indicators that indicate a “brain at risk” in vascular brain damage, opening up a possible window of time for early identification and assistance for people who run the risk of experiencing cognitive decline [80].

### 2.5. Biofeedback

Biofeedback, rooted in operant conditioning from psychological learning theory, is a therapeutic method that teaches individuals to recognize and control specific physiological functions [81]. This process enables patients to perceive and regulate their internal state or external performance, aiding in the recovery of bodily or mental functions post-trauma and contributing to an overall improvement in well-being. Operating within a self-contained, self-regulatory loop, biofeedback systems gauge the individual’s physiological state, process the gathered data, and then relay this information through instructive signals. This process underscores the significance of reflex-triggering events and feedback connections in molding physiological functions across diverse levels [82]. This includes information on heart rate, muscle tension, and skin temperature. Conditions like stress, anxiety, chronic pain, and certain neurological disorders can compromise immune responses; however, techniques such as imagery and relaxation, administered through biofeedback-assisted relaxation, have been proven to bolster immune function. This is particularly evident through the increase in phagocytic activity among individuals initially experiencing high stress and low phagocytic capacity [20,83]. Eight sessions of biofeedback therapy dramatically reduced felt stress and EMG levels while enhancing academic resilience, according to a new study involving 34 senior nursing students. These findings imply a possible connection between stress-reduction techniques and the interaction of the immunological and neurological systems [84]. Another study investigated the effects of biofeedback-based progressive muscle relaxation on stress in first-year Korean nursing students in their clinical rotation. In comparison to the control group, the experimental group exhibited significant reductions in NK cell count stability, blood pressure levels, and stress symptoms [85]. The effect of biofeedback-assisted methods has been investigated further: a pilot investigation with rheumatoid arthritis patients revealed significant decreases in the rheumatoid factor, pain behavior, and self-reported pain intensity following individual thermal biofeedback training sessions and cognitive-behavioral group therapy, suggesting potential benefits for autoimmune and pain-related conditions [86].

## 3. Therapeutic Approaches Addressing the Neuroimmune Interface in Clinical Pain Management

A substantial health issue, chronic pain, is estimated to have affected 50 million US individuals (20.4%) between 2016 and 2019, accounting for up to 60% of ER visits due to pain-related issues. Wide-ranging effects of this problem include an annual loss of productivity of roughly $61 billion along with medical expenditures for chronic pain that exceed the total expenses of heart disease and cancer by $560 to $635 billion [87,88]. Despite these limitations, many patients with chronic pain experience poorly controlled pain as a result of the present method it is managed with, which contributes to the ongoing opiate issue [89]. It is important to understand the reciprocal interactions that occur among neurons and the immune system in order to understand the origins of chronic pain. Immunogenic inflammation causes nociception, but neurogenic inflammation can activate both the adaptive and innate immune systems. Sustained neuroinflammation has been connected to chronic pain syndromes, such as chronic migraines with elevated concentrations of peptides related to the calcitonin gene (CGRP) [90].

### 3.1. Modulation of the Neuroimmune Interface by Anti-Inflammatory Agents

Treatments that target specific pro-inflammatory signaling pathways have shown promise in the therapeutic management of inflammatory pain, particularly in the context of diseases such as ankylosing spondylitis or rheumatoid arthritis (RA)-related lower back pain [91,92,93,94]. Inhibitors of the IL-1b/IL-1R signaling pathway, such as anakinra, riloncept, and canakinumab, have proven efficacy and good tolerability in various inflammatory diseases involving IL-1b deregulation [95]. Clinical trials show that patients with RA treated with anakinra experience significant clinical benefits, reduced inflammation markers, and slowed joint damage progression [96]. In a preliminary study treating an acute anterior cruciate ligament injury to the knee, anakinra also demonstrated promise [97]. According to a mice model for chronic regional pain syndrome (CRPS), neuropathic pain may be treated with IL-1 antagonists, according to recent research [98].

Analogously, anti-TNFα antagonists such as adalimumab, etanercept, and infliximab are used to treat inflammatory bowel disorders and RA by reducing pain and associated symptoms [99,100,101,102,103]. Their combined effectiveness with methotrexate is especially strong in treating RA. Anti-TNFα therapy is not commonly employed in routine pain management, despite some evidence to the contrary [104,105,106].

Monoclonal IL-6 receptor inhibitors such as sarilumab, satralizumab, and tocilizumab, along with the IL-6 sequestering antibody siltuximab, target IL-6, which is an additional irregular pro-inflammatory cytokine in chronic pain syndromes [107,108,109,110]. These IL-6 antagonists are clinically effective, with ongoing research exploring their potential applications in lower back pain [111,112,113].

Research on the efficacy of CGRP antagonists in treating other pain syndromes, like trigeminal neuralgia and fibromyalgia, has been spurred by their success in preventing migraine headaches [114,115]. Despite these successes, not all drugs targeting neuroimmune signaling have translated well into clinical practice. For instance, CCR2 antagonist (AZD2423), TLR4-blocking antibody (NI-0101), and P2X7 purinergic receptor antagonist (AZD9056) showed no clinical benefit in specific studies [116]. However, the potential applications of these drugs in various pain conditions, along with many other drugs targeting the neuroimmune interface, remain to be explored.

### 3.2. Electrical Stimuli in Chronic Pain Management

A new discipline of neuromodulation uses electrical stimulations as a pain management strategy for treating chronic pain [117]. The therapies include dorsal root ganglion excitement, peripheral nerve stimulation, brain stimulation, and spinal cord stimulation (SCS). Understanding the role of non-neuronal activity, including glial cells, helps us comprehend the mechanisms behind neuromodulation.

SCS is a commonly used neuromodulation therapy that has been shown to be beneficial in several neuropathic pain syndromes, including complicated regional pain syndrome, diabetic neuropathy, and post-laminectomy pain syndrome [118,119,120]. Following a variety of nerve lesions, rodent models receiving SCS exhibit decreased pain behavior, which is consistent with a decrease in glial activation markers and transcriptome changes in genes related to immunological response and neuroinflammation [121,122,123]. A unique SCS waveform that was designed based on preclinical tests to coincide with transcriptome profiles of neuronal and glial populations is an example of a successful bench-to-bedside translation. The transcriptome characteristics that emerge are like those of undamaged, naïve profiles. In a multicenter clinical study, this waveform performed better than conventional waveforms, obtaining a response rate of 80% in patients with chronic back pain [124,125,126,127].

Vagal nerve stimulation has been previously discussed as a possible method in the context of neuromodulation. By inhibiting excessive cytokine release and inflammation through its signaling pathways, the vagal nerve, which has traditionally been used to treat resistant depression and refractory epilepsy, leads to an inflammatory response [128,129]. Patients with epilepsy who receive vagal nerve stimulator treatment have lower periphery TNFα, IL-1b, and IL-6 levels [130]. The non-invasive transcutaneous vagal nerve stimulator, which was first developed to treat acute migraines, has demonstrated efficacy in decreasing pro-inflammatory cytokines in peripheral blood. Vagal nerve stimulation is currently a successful treatment for a number of other inflammatory/autoimmune diseases, including Crohn’s disease, rheumatoid arthritis, and COVID-19 [131,132,133].

### 3.3. Steroid Injections in Pain Management: Unlocking the Potential of Epidural Glucocorticoids

A key component of interventional pain therapy is the administration of steroids in or near the sites of pain, with epidural glucocorticoid injections being a popular technique for treating persistent spinal pain. Knowing that more than two million lumbar epidural glucocorticoids are given yearly just to Medicare beneficiaries shows how widely used the medication is. This method has demonstrated promise in lessening the severity of pain, decreasing the need for opioids, enhancing function, preventing surgery, and even controlling pain when surgical interventions are not successful [134,135]. The epidural steroids dexamethasone, triamcinolone, methylprednisolone, and betamethasone may have anti-inflammatory impacts on the neuroimmune interface.

In line with preclinical model findings, steroids given locally, intrathecally, or systemically prior to or during injury have shown promise in reducing inflammatory cytokines, neuronal firing rates, and glial cell stimulation in the spinal cord. Following nerve damage, there is a correlation between this drop and a decrease in pain behavior. Interestingly, steroids do not affect anti-inflammatory cytokines like IL-4 and IL-10, which similarly decrease after injury, even if they successfully lower pro-inflammatory cytokines after injury. This implies the presence of an anti-inflammatory mechanism independent of steroids.

Notwithstanding these advantages, the efficacy of steroids in reversing established pain behavior in animal models is not entirely consistent. Similarly, the application of epidural steroids to treat spinal pain is not well-supported by clinical data. Steroids have the ability to stimulate pro-inflammatory mineralocorticoid receptors in sensory neurons of the dorsal root ganglia (DRG). Steroids are frequently used in medical therapy. When a mineralocorticoid receptor antagonist is administered in addition to dexamethasone in mouse models of lower back pain, the effects are greater in reducing both evoked and spontaneous pain responses as well as the stimulation of satellite glial cells (SGCs) within the DRG. The variable effectiveness of steroids in clinical practice over time may be attributed to the varied activation of steroid receptors. Given the extensive use of mineralocorticoid receptor antagonists, such as spironolactone and eplerenone, in the treatment of hypertension and heart failure, this presents exciting opportunities for future clinical research [136].

### 3.4. Glial Inhibitors/Modulators and Their Impact on the Neuroimmune Interface

Glial cells, primarily astrocytes, microglia, and oligodendrocytes play crucial roles in the CNS beyond just providing structural support. They are key modulators of the neuroimmune axis that involve interactions between neurons, glial cells, and immune cells, integrating immune responses with neuronal function. Glial inhibitors and modulators are pharmacological agents designed to selectively target glial cell functions and, consequently, modulate neuroimmune responses. Their aim is to suppress detrimental glial activation (e.g., in neurodegenerative diseases, chronic pain, and neuroinflammation) or enhance protective glial functions. Glial inhibitors and modulators represent a promising therapeutic strategy by precisely targeting the complex roles of glial cells in the neuroimmune axis. Their ability to inhibit detrimental glial activation or enhance protective responses can modulate neuroinflammation, synaptic function, and overall CNS health, offering potential benefits in various neurological and neurodegenerative conditions. Numerous medications that were able to block glial function in preclinical research have not been shown to be useful in clinical settings. Minocycline, a semi-synthetic tetracycline that has been widely used for over thirty years, has anti-inflammatory, anti-apoptotic, as well as anti-angiogenic properties in addition to its antibacterial efficiency. Perhaps most significantly, though, is its ability to inhibit microglial activation [137,138,139]. Clinical data are conflicting and weak, despite several preclinical studies supporting minocycline’s beneficial effects in reducing pain behavior in neuropathic pain models. Research conducted in a variety of pain contexts revealed that minocycline either did not offer clinically significant advantages or, in some patient populations, prolonged the duration of pain [136,140].

The hypothesis that the stimulation of microglial cells is the primary cause of pathogenesis in different medical distress environments, the low number of participants in the clinical studies, and the possibility that minocycline targets non-microglia could be the reasons for the discrepancy between preclinical and clinical outcomes [139]. Propentofylline, a general microglia and astrocyte glial inhibitor, demonstrated promising preclinical results in the prevention and treatment of neuropathic pain in a randomized clinical study [141,142]. However, propentofylline did not appear to be effective in treating post-herpetic neuralgia patients.

Ibudilast, a cyclic nucleotide phosphodiesterase (PDE) inhibitor that is non-selective and is used as a bronchodilator for asthma, has glial inhibitory effects by inducing activated microglia to produce IL-10 and inhibiting TNFα, IL-1β, and IL-6 production [143]. Despite lacking efficacy in clinical trials for CRPS, diabetic neuropathy, or chronic migraines, ibudilast shows potential as a treatment for substance use disorders, including stimulant, alcohol, and opioid usage [144,145,146,147]. However, in an additional analysis of PROMISE-2 patients with a combined condition of medication-overuse headaches and chronic migraine, eptinezumab demonstrated notable efficacy, providing information about the drug’s potential advantages for this population. Furthermore, the investigation assessed the safety and tolerability of eptinezumab in these individuals, offering significant new perspectives on managing persistent migraines with co-occurring medication-overuse headaches [148].

### 3.5. The Role of Vagus Nerve Stimulation in Alleviating Chronic Pain Conditions

In recent decades, both animal and clinical investigations have indicated the potential analgesic impact of vagus nerve stimulation (VNS) under specific parameters. A rising corpus of research examining the function of VNS in pain management has resulted from the increasing availability of non-invasive VNS (nVNS). Traditionally used for conditions like epilepsy and depression, VNS is now gaining attention for its potential analgesic effects under specific parameters. This interest has been further fueled by the increasing availability and accessibility of nVNS devices, which provide a safer and more convenient alternative to the surgically implanted versions. VNS, especially in its non-invasive form, is reshaping the modality of pain management in clinical practice. By offering a novel, effective, and patient-friendly approach to pain relief, nVNS is enhancing the quality of care and opening new possibilities for treating chronic pain, marking a significant advance in both clinical practice and patient empowerment [149].

#### 3.5.1. Vagus Nerve Stimulation in Chronic Widespread Pain: Modalities and Efficacy

It has been shown that VNS can regulate nociception and treat a range of clinical pain disorders. Conditions such as fibromyalgia, which is linked to fatigue, sleep disturbances, depression, and cognitive dysfunction, fall under the category of chronic widespread pain that is marked by major affective problems and dysfunction. Following 11 months of treatment, five of the patients in Lange et al.’s original open-label research on the effectiveness of invasive VNS (iVNS) for fibromyalgia no longer met diagnostic criteria [150]. Kutlu et al. investigated the effects of transcutaneous VNS (taVNS) in conjunction with exercise in patients with fibromyalgia due to the intrusive nature of iVNS and found that there was a substantial decrease in pain intensity [151]. Nevertheless, more studies with bigger sample numbers and a range of stimulation settings are necessary to completely comprehend how nVNS affects widespread chronic pain.

#### 3.5.2. Chronic Trigeminal Allodynia

Sodium channel blockers are commonly used to treat trigeminal neuralgia, and surgical and radiotherapy therapies become alternatives when non-conventional medical treatments fail. Oshinsky et al. examined the potential of transcutaneous vagus nerve stimulation (tcVNS) in a rat model and found that it prevented glutamate rise and reduced sensitivity in the trigeminal nucleus caudalis. These results open up a promising new direction, but more clinical research is necessary to confirm that tcVNS is effective in treating trigeminal neuralgia patients [152].

#### 3.5.3. Chronic Musculoskeletal Pain: Integrating Physiological Modulation and Vagus Nerve Stimulation

Chronic musculoskeletal pain, defined as persistent or recurrent discomfort originating from conditions damaging the skeleton, joints, muscle tissue, or related soft tissues, requires a comprehensive strategy to deal with both signs and underlying conditions [153]. Rehabilitation, exercise, and interventional treatments are among the non-pharmacological pain management approaches used to assist in alleviating pain to some extent, in addition to commonly used analgesics such as relaxation drugs, opioids, anticonvulsants, and antidepressants. In research by Frøkjaer et al., deep breathing to boost vagal tone and transcutaneous vagus nerve stimulation (taVNS) effectively reduced somatic pain sensitivity and raised thresholds for pain in musculoskeletal areas in healthy participants [154]. The anti-inflammatory properties of VNS help it even more effectively cure chronic musculoskeletal pain.

Immune system issue preclinical data demonstrated that vagotomy-affected rats had worsening rheumatoid arthritis, which is a chronic autoimmune inflammatory disorder that destroys and inflames joints [155]. Preliminary research indicates that invasive stimulation of the vagus nerve improved the way rheumatoid arthritis patients were evaluated for pain [156]. According to studies by Venborg et al., tcVNS dramatically decreased hip pain in individuals suffering from polymyalgia rheumatica, a disorder characterized by persistent stiffness and pain in the muscles [157]. TaVNS therapy for systemic lupus erythematosus considerably decreased pain and fatigue during a 12-day period [158].

Regarding osteoarthritis, in a study by Krusche-Mandl et al., electric auricular acupuncture reduced pain and increased the amount of time patients could walk without experiencing any pain [159]. Even after six weeks of a nonstop, small amount of electrical auricular acupuncture, sustained effects were seen during follow-up, suggesting a potential role for taVNS in osteoarthritis.

When it comes to medication, treating persistent back pain caused by lumbar spine abnormalities is challenging, and non-pharmacological methods are not very effective [160,161]. Continuous auricular electroacupuncture has been shown by Sator-Katzenschlager et al. to be a successful method of pain relief for people with persistent lower back pain [162,163]. Additionally, a pilot trial showed that the combination of mindful meditation with taVNS reduced the intensity of back pain and raised the threshold for pressure discomfort. These results suggest that VNS might be a helpful low-back pain treatment.

#### 3.5.4. Challenges and Future Directions in Vagus Nerve Stimulation for Chronic Pain Management

Virtual neural stimulation (VNS) presents itself as a potentially effective neuromodulation treatment option for chronic pain syndromes, offering a non-pharmacological alternative with fewer side effects. Nevertheless, there are certain drawbacks to the existing research on VNS for the treatment of chronic pain, including short intervention times that produce inconsistent outcomes, limited sample sizes, and a dearth of investigation into the best demographics for VNS. Future research should include larger-scale, longer-term randomized controlled studies to validate current findings in order to solve these issues. Additionally, it ought to investigate the use of VNS in a range of chronic pain situations, enhance stimulation settings, and pinpoint patient types who are most likely to react well. Moreover, additional investigation is required to identify the specific neural pathways and mechanisms responsible for the analgesic effects of VNS [164].

## 4. Neuroimmunomodulation in Aging

An important environmental component of aging is its association with several neurodegenerative illnesses as well as “inflammaging”, a state marked by low-grade systemic inflammation [165]. Recurrent activation of astrocytes and microglia throughout an individual’s life causes damage from free radicals, oxidative stress, and mtDNA buildup, ultimately resulting in a ‘primed’ phenotype [166]. An increased baseline inflammatory state, a heightened pro-inflammatory response to stimuli, and a decreased ability to maintain homeostasis are the symptoms of this priming. Systemically, inflammation causes the blood to produce inflammatory mediators at low levels, including TNF-α, CRP, and IL-6, which exacerbates the inflammatory milieu in the central nervous system [165,167]. Additionally, studies indicate that BBB permeability is higher in older animals, which facilitates peripheral immune cells’ entry into the central nervous system. Additionally, studies indicate that BBB permeability is higher in older animals, which facilitates peripheral immune cells’ entry into the nervous system’s nerve cells. To completely comprehend the precise molecular causes of inflammation, further research is essential. In myeloid cells from aged mice, greater amounts of the lipid transmitter prostaglandin E2 (PGE2) were linked to worse bioenergetics. An energy-deficient state and maladaptive pro-inflammatory reactions follow from this. Aged mice’s cognitive function was sufficiently restored by inhibiting peripheral myeloid PGE2 signaling, demonstrating the possibility of reprogramming glucose metabolism to reverse dysregulated immunological activities. Myeloid cell glucose metabolism has emerged as a potential target for therapy [166].

Inflammaging plays a significant role in cognitive decline and the progression of neurodegenerative disorders like Alzheimer’s disease (AD) and Parkinson’s disease (PD). It is driven by factors such as cellular senescence, where aging cells secrete pro-inflammatory molecules known as the Senescence-Associated Secretory Phenotype (SASP). In the brain, inflammaging causes microglia to become overactive, releasing inflammatory molecules that damage neurons and synapses, thus accelerating cognitive decline. Additionally, immunosenescence—the aging of the immune system—reduces its ability to combat infections while increasing chronic inflammation, further promoting neurodegeneration. This inflammatory environment hastens neurotoxic events; in AD, it accelerates amyloid-beta plaque and tau tangle formation, while in PD, it leads to the loss of dopaminergic neurons in the substantia nigra, causing motor, cognitive, and mood symptoms [165,166,167,168,169,170].

### 4.1. Neuroimmune Communication in Adulthood and Aging: Insights and Health Implications

There is a mutual relationship among the immunity system as well as the brain and spinal cord that lasts into maturity. As part of an illness response, peripheral immune cells that are activated by infection frequently release cytokines that promote inflammation and other mediators. This pro-inflammatory signaling enters the central nervous system through passive diffusion, direct neuronal transmission, and regulated passage across the blood–brain barrier. Microglia along with other neuroimmune cells secrete pro-inflammatory cytokines once they reach the central nervous system, which results in transient neuroinflammation and disease-like behaviors [168]. Neuroimmune reactions in healthy individuals are often predisposed to anti-inflammatory reactivity, which facilitates effective immune resolution [169,170].

The BBB is one example of a CNS-immune interface. Its special properties help to minimize excessive immune signals while promoting communication. Immune cells cannot enter the CNS unhindered because of the BBB’s endothelial barrier, which tightly regulates the movement of cells and solutes [171]. Nevertheless, in response to peripheral immunological signals, immune cells located in meningeal compartments, such as macrophages, T cells, and B cells, can generate immunomodulators [5,172].

As people age, their immunological and neuroimmune systems become less effective; this decline begins in middle age and picks up speed in later life. As the body’s immune system matures, both adaptive and innate immune cells show decreasing sensitivity and variety, making it more challenging to identify and eradicate infections. Seniors’ central nervous systems (CNSs) also show changes in immune responsiveness, with microglia exhibiting neuroimmune priming that exacerbates pro-inflammatory responses. Age-related alterations, such as elevated blood–brain barrier permeability and a pro-inflammatory slant in meningeal immune cells, intensify immune-to-CNS communication. These changes in neuroimmune responsiveness with aging lead to extended and hypervigorated neuroimmune responses, which may have long-term negative effects on behavioral and physiological processes [170,171,172,173,174,175].

### 4.2. Inflammaging: The Complex Interplay of Pro-Inflammatory Mechanisms in Aging

“Inflammatory cytotoxicity”, an overall pro-inflammatory illness, is defined as an imbalance among pro- and anti-inflammatory mechanisms that leads to increased cytokine production. This imbalance results in a protracted state of low-grade inflammation that raises pro-inflammatory mediators such as IL-1b, IL-6, TNF-a, IL-8, and CRP [176]. This phenomenon is thought to be a biomarker of accelerated aging and is a characteristic of aging [177]. Inflammation is influenced by several interconnected processes; at the physiological level, weight gain, a lack of exercise, emotional strain, early-life adversity, xenobiotic exposure, and chronic infections are some relevant variables that contribute to inflammation. Inflammation is also known to be a risk factor for a wide range of pathologies, including viral diseases, depression, cancer, sarcopenia frailty, and cardiac, renal, and neurological disorders [177,178,179,180].

Moreover, a number of studies associate inflammation with a higher likelihood of severe COVID-19 issues in the elderly. They attribute this to an overreaction to the virus that results in a large-scale release of chemical mediators [177,179,181,182]. According to new theories, inflammation-aging is an adaptive process that, depending on lifestyle, environmental, and genetic factors, can result in either a pathological state or healthy aging [176,180]. Research on centenarian communities supports this idea by showing that high levels of inflammatory biomarkers interact with anti-inflammatory chemicals to prolong life [183]. The process of inflammation is dynamic and multifaceted, involving multiple age-related molecular pathways that go beyond a direct relationship with the immune system [180]. For instance, oxidative stress causes age-related transcriptional changes in genes that encode crucial components of inflammatory pathways. Senescent cells’ pro-inflammatory secretome can paracrine affect surrounding tissues, sustaining the inflammatory state throughout the organism [176]. The last major factor contributing to inflammaging is the dysregulation of the microbiome; it is thought that treating age-related dysbiosis with probiotics could reduce inflammaging [178,184].

### 4.3. Neuroinflammatory Landscape in Aging: A Glial Perspective

The CNS experiences significant changes in its inflammatory state with age, including increased oxidative stress, decreased neurogenesis, a higher risk of region-specific loss and neurodegeneration, and an overall increase in inflammatory tone. The aging brain’s elevated inflammatory tone is caused by a variety of cell types, including neurons, glial cells (including astrocytes, microglia, oligodendrocytes, and ependymal cells), immune cells that have infiltrated the area, and nonglial CNS-resident cells (perivascular macrophages, pericytes, as well as endothelial cells). Crucial to the dynamic neuroimmune milieu are glial cells, of which age-related neuroimmune alterations include pro-inflammatory phenotypes and neuroimmune priming. These neuroimmune alterations associated with healthy aging could be defensive or compensating processes in response to the system’s slow deterioration. Regrettably, CNS injury and susceptibility may also rise as a result of these aging-related neuroimmune changes [185].

### 4.4. Microglial Changes in Aging: A Pro-Inflammatory Shift with Regional Variations

Microglia grow increasingly dysfunctional and pro-inflammatory as we age. The expression of inflammatory genes such as Spp1, Itgax, Axl, Lgals3, Clec7a, Trem2, and Cd68 rises with microglia age, while the expression of homeostatic microglia effectors falls. Microglia develop into diverse populations as they mature, some of which show inflammatory markers (such as chemokines Ccl4 and Ccl3) including the pro-inflammatory cytokine IL-1β that is specific to macrophages [185,186]. The CNS may be more easily reached by immune cell populations thanks to this increase in chemokine communication. Moreover, inflammatory microglia age less effectively than adult microglia and frequently remain in a pro-inflammatory state for a significantly longer period [187].

The microglia in the aging brain show a range of functional alterations. Mice (thirteen months old) show regional heterogeneity in the age-associated microglia phenotype starting in middle age. Microglia proliferate in many brain regions as individuals age. The cortical, hippocampal, CNS white matter networks and basal ganglia elements are among these areas [188]. By the location of the brain, microglia also have different transcriptional signatures. Microglia in elderly white matter produce cell states associated with activation and phagocytic clearance of degenerating myelin, whereas microglia in the aged gray region primarily operate in homeostatic cell states [189]. Regional sensitivity is further supported by proteomic investigations. While the aging brain as a whole experiences metabolic alterations, the extent of these changes varies depending on the region [190]. The CNS inflammatory milieu probably has an impact on aging-related alterations in microglia function. Aging can, in fact, change the CSF proteome, which can affect the phenotypic and function of microglia [191,192].

### 4.5. Aging-Associated Transformations in Astrocytes: Implications for Neuroinflammation

The majority of glial cells in the central nervous system, known as astrocytes, perform a variety of vital tasks for maintaining CNS homeostasis, including controlling lymphatic function, altering synaptic transmission, fortifying the blood–brain barrier, providing physical support, and releasing chemokines, cytokines, and neurotrophic molecules [193]. However, astrocytes undergo significant phenotypic and functional alterations as the aging process progresses.

As we age, astrocytes proliferate, which could relate to a neuron-to-glia fate flip that encourages astrocytic development. The age-related cognitive declines may be exacerbated by alterations in hippocampal neurogenesis. Additionally, there are regional differences in the vulnerability of astrocytes, with the hippocampus, hypothalamus, and cerebellum being among the brain regions with higher reactive states [193,194,195,196]. This increased sensitivity is more noticeable in older astrocytes (>20 months in mice), which is like characteristics seen in conditions like Alzheimer’s [197]. Furthermore, aging astrocytes show changes in both their morphology and functionality. They have smaller territorial domains (depending on area), fewer processes, and lower intercellular connectivity morphologically. Aged astrocytes function less efficiently in glutamate absorption and potassium elimination. Impaired synaptic plasticity is correlated with a decrease in astrocyte activity in the aged brain [198].

As we age, so do the communication patterns between astrocytes and microglia, the central nervous system’s major immune cells. Pro-resolution phases of microglia are disrupted by aging, and these states are critically dependent on astrocyte-derived anti-inflammation and cholesterol synthesis pathways [187]. The complex interactions between both kinds of cells are shown by the way that microglia induce more reactive phenotypes in astrocytes [194]. With age, gender variations in the neuroimmune environment become apparent, with females showing a lower phagocytic signature and a more pro-inflammatory baseline [199,200]. These gender-specific reactions could be the cause of the differences in neuroinflammatory disease susceptibility between both genders.

In addition to inherent changes, outside factors also stimulate inflammatory cells in the aged central nervous system. Age-associated microglial priming is influenced by a build-up of damage-associated molecular patterns, heightened permeability of the blood–brain barrier, and compromised waste clearance systems [201,202,203,204,205,206,207].

Indeed, astrocytes undergo complex alterations as we age, which affect their quantity, morphological characteristics, regional responsiveness, and functional abilities [193]. Complex alterations in microglia–astrocyte interactions occur, contributing to the pro-inflammatory milieu observed in the aging central nervous system. Understanding the processes behind age-associated neuroinflammation and its consequences on the functioning of the brain requires deciphering these complex alterations.

### 4.6. Impact of Age-Related Shifts in Meningeal Immune Cells on CNS Function

Immune cells outside of the CNS substructure have a major effect on the way the CNS functions during an organism’s life. These cells are found in regions that are outside of the blood–brain barrier, including the meninges and choroid plexus, and they are involved in the intricate regulation of neuroimmunity. The meningeal and choroid plexus compartments comprise an extensive range of immunity cell types, such as monocytes, cells known as dendritic cells, B cells, T cells, and natural killer cells, among others [171]. This is in contrast to adult animals, whose healthy parenchyma has a relatively modest amount of immune cell types. These bone marrow-derived and refilled meningeal immune cells in the surrounding skull and vertebral bodies can originate and spread neuroimmune signals, which may penetrate the CNS parenchyma under pathological circumstances [166].

Peripheral immune cells in the meninges affect complex activities including social behavior and cognition because of their significant function in neuroimmunity. CD4+ T-cell reduction impairs memory and long-term potentiation. T cells have been identified as important modulators of learning and memory. The complex mechanisms regulating cognitive functioning are highlighted by the interaction between T cells and GABAergic neurons, which is mediated by IL-4-dependent signaling [208]. Moreover, T-cell signaling from the meningeal compartment controls neuronal GABAergic activity and contributes to changes in social behavior, especially through interferon (IFN)-γ/JAK-STAT [209]. The significance of meningeal γδ T cells and IL-17 in short-term memory illustrates the complex connection that exists between immune system cells and cognitive processes [18]. Age has a major impact on the makeup and function of the meningeal compartment T-cell population. Increased T-cell counts as well as changes to T-specific subpopulations foster a persistent pro-inflammatory slant. This age-related shift in the meningeal T-cell landscape is associated with aging-related CNS impairment. Studies employing Rag1-/-deficient T-cell-deficient animals demonstrate resilience to age-related deterioration as well as reductions in locomotor and cognitive abilities. Furthermore, behavioral changes and age-related axon deterioration are associated with changes in the proportions of meningeal T-cell subpopulations, specifically cytotoxic CD8+ T cells [166].

### 4.7. Leveraging Peripheral Immunity to Mitigate Neuroinflammation in Aging

Peripheral immunity and the central nervous system interact to provide a strategic means of adjusting the neuroinflammatory consequences of aging. It is interesting to note that age-related alterations in microglia are influenced by the gut microbiome. Microbiota transfer has been shown to modify the aged gut microbiota, reducing age-related alterations in the neuroimmune milieu and improving cognitive function [210,211]. Fetal microbiota transfer may enhance neuroimmune states by reducing age-related elevations in δ-valerobetaine, a metabolite produced from gut microbiota and present within the blood and the central nervous system. Remarkably, microglia might not be required for the beneficial effects of fetal microbiota transplantation [212]. Adding commensal environmental bacteria, such as *Bifidobacterium adolescentis*, has also been demonstrated to increase host metabolism and catalase activity, as well as prolong lifespans and enhance overall health in a range of animals. Moreover, the commensal bacteria Mycobacterium vaccae protects from age-associated neuroinflammation and afterwards cognitive loss in aged rats that have been vaccinated. By modifying T-cell–CNS signaling, Mycobacterium vaccae treatment may lessen microglial priming [173,213]. Taken together, these results show the potential for “rescuing” peripheral immunological signals during the aging process to lessen age-related changes in the neuroimmune milieu.

### 4.8. Age-Related Alterations in T- and B-Lymphocyte Function and Transcriptional Regulation

T lymphocytes have functional changes that affect their function as people age. T-cell numbers have been shown to decline with age, and replicative senescence caused by telomere shortening has been shown to reduce proliferation [214]. Furthermore, older people frequently have higher numbers of T cells that are positive for beta-galactosidase activity linked with senescence [215]. Chronic inflammation throughout aging is associated with this buildup of immunosenescent T cells [216]. Chronic infections and worn-out, non-functioning T cells combine to cause hyperinflammatory conditions [217]. Two essential transcription factors, transcription factor 7 (TCF7) and thymocyte selection-associated high-mobility group box (TOX), regulate the development of T cells from the point of exhaustion onward. The HMG box DNA-binding protein family member TCF7 is essential to the development and maturation of T-lineage cells. Establishing the WNT/β-catenin signaling pathway with β-catenin, it promotes the expression of genes linked to both adult stem cell self-renewal and embryonic development [218,219]. T lymphocytes with an exhausted phenotype during long-term viral infections have TCF1 present, which gives them the capacity to endure, self-renew, or multiply [220]. In contrast, persistent activation of CD8+ T cells activates TOX, which is mostly expressed in hematological and immunological organs, especially in CD4+ T and natural killer cells [221]. Through chromatin remodeling and the activation of T-cell inhibitory receptors, such as protein disulfide isomerase, TOX activity facilitates CD8+ T-cell fatigue [222].

**Figure 3:**
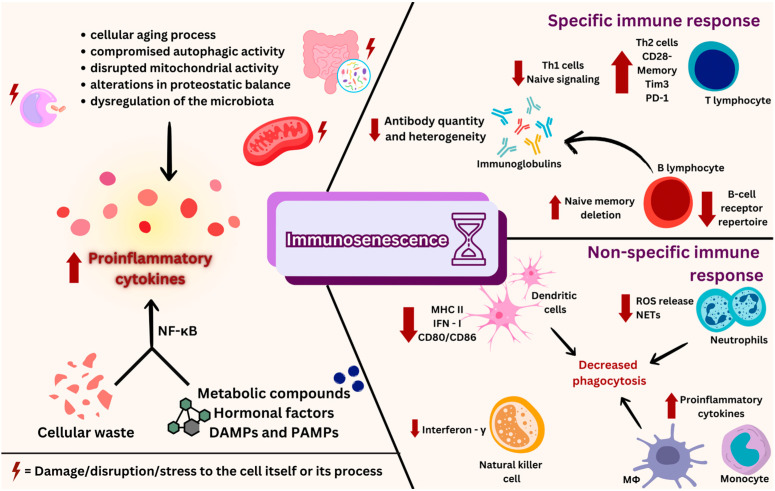
The most common form of neuroinflammation in aging is a chronic low-grade inflammatory condition in the brain and spinal cord. Reactive oxygen species and cytokines, two inflammatory mediators, are present in higher concentrations throughout this process. Contributing factors include senescent cell-induced secretory phenotypes, sterile components from cell cycle-related debris, and the impact of chronic infections. In aging brains, the diminished ability to resolve inflammation and the accumulation of neurotoxic molecules exacerbate this condition. Neuroinflammation may disrupt neural function, potentially contributing to age-related cognitive decline and neurodegenerative diseases. (The up and down arrows in the image represent the increase (up arrow) or decrease (down arrow) of the processes or factors they are next to, such as increasing proinflammatory cytokines or decreasing phagocytosis).

B lymphocytes, which produce antibodies and are in charge of humoral immune responses, help to differentiate between self and non-self antigens. They also help to create memories of past pathogen contacts, which can result in an improved response in future host–pathogen interactions [223]. A population of atypical defective B cells, which are incapable of differentiating into cells that produce antibodies, accumulates after long-term viral infections. These B cells also exhibit a decreased ability to stimulate the generation of cytokines and antibodies, as well as the activation of B-cell receptors [224,225]. On the other hand, unlike the continuous T-cell response that eventually wears out, B-cell responses within germinal centers continue to be strong and effective as the infection worsens [226]. An excessively pro-inflammatory milieu is created by the continuous immune response, and B cells produce more autoantibodies in this context. The onset of immunosenescence, which reflects alterations in B-cell aging and affects elderly people’s defenses against infections, is substantially aided by the creation of this inflammation-feedback loop, as presented in Figure 3 [227,228].

### 4.9. Therapeutic Approaches for Mitigating Immunosenescence and Inflammaging: A Multifaceted Strategy

As we age, our immune systems become more dysfunctional and hyperactive, both in terms of innate as well as adaptive responses. This mechanism plays a part in the emergence of inflammatory chronic diseases that are common in elderly people [229]. In response to these challenges, an abundance of pharmacological and cellular/genetic strategies have been developed to mitigate or even reverse the deleterious consequences of immunosenescence on health [230]. These tactics cover a variety of methods, including (a) using induced pluripotent stem cells (iPSC) to produce targeted immune cells and hematopoietic cells; (b) increasing macrophage activity by administering growth factor and cytokine cocktails; (c) bone marrow transplantation, which is a commonly used treatment to regenerate the thymus [231]; (d) using Cdc42 and basic leucine zipper transcription factor (BATF) inhibitors or antioxidants to increase the quantity and capacity of lymphoid-biased hematopoietic stem cells [232,233]; (e) boosting memory CD4+ T-cell function through the inhibition of dual-specific phosphatases 4 [234]; (f) using fibroblast growth factor 7 (FGF7) to stimulate the creation of naïve T cells and aid in the elimination of unhealthy cells in order to restore thymus function [235]; and (g) improving CD8+ T-cell function through the administration of rapamycin [236,237].

Calorie restriction is another important non-pharmacological tactic that has been shown to strengthen immunity. Through the activation of insulin-like growth factor 1 (IGF-1) and/or peroxisome proliferator-activated receptor (PPAR) pathways, this strategy enhances thymopoiesis and delays the generation of senescent T cells [238,239]. Functional foods may help reduce inflammation and oxidative stress while also enhancing lipid metabolism, which is linked to metabolic disorders, according to recent research. The NF-kB and/or Nrf2 signaling pathways are responsible for these effects [240,241]. There is therapeutic potential in some molecules and mechanisms that affect immunosenescence. In an effort to reduce inflammation, the activator protein 1 (AP-1) signaling pathway, which is essential for macrophage-mediated inflammation, has been targeted. For example, systemic and hepatic inflammation was reduced in mice given a high-fat diet when lentiviral small interfering RNAs (siRNA) targeting AP-1 were transfected [242]. Furthermore, rosiglitazone, a PPARg agonist, showed promise in treating sepsis in mice by lowering cardiac inflammation and cell death. Improved insulin resistance and enhanced fatty acid oxidation were seen in human skeletal muscle [243]. Therapies intended to reduce inflammation must concentrate on the synergistic effects of numerous substances, concurrently regulating distinct pathways, due to the complexity of aging involving many biological processes. As an instance, a combination therapy including three distinct substances—rapamycin, acarbose, and 17a-estradiol—converges on controlling the p38-MAPK and ERK1/2 pathways [244].

## 5. Neuroimmune Dynamics in Mood Disorders

The relationship between the immune system and mood disorders, such as depression and anxiety, is a dynamic and complex interplay. This bidirectional interaction suggests that mood disorders are not solely psychological or neurological but are deeply entangled with immune system functions. On the one hand, mood disorders often coincide with a state of chronic low-grade inflammation. Individuals with depression, for instance, frequently show elevated levels of pro-inflammatory cytokines—such as IL-1β, IL-6, and TNF-α—in their blood and cerebrospinal fluid. In the brain, these cytokines can alter neurotransmitter systems, particularly serotonin, dopamine, and glutamate, which are critical for mood regulation. The result is a disruption in neural circuits that regulate emotions, leading to symptoms like persistent sadness, anxiety, and cognitive impairments. Conversely, mood disorders themselves can influence immune function. Psychological stress can activate the hypothalamic–pituitary–adrenal (HPA) axis, leading to the release of cortisol. While cortisol has anti-inflammatory effects in acute situations, chronic stress can lead to cortisol resistance, where immune cells become less responsive to its regulatory effects. This condition exacerbates inflammation, creating a vicious cycle where the immune system remains in a heightened state of activation, further aggravating mood symptoms [245,246,247,248].

Mood disorders continue to be a challenge in the field of mental health, even in the face of significant advancements in psychiatry. These conditions collectively exert a negative impact on individuals’ psychological well-being, with major depressive disorder affecting a substantial portion of adults, ranging from 3% to 17%, and bipolar disorder (BD) prevalent in approximately 1% to 3% of the general population. Simultaneously, mood disorders share the stage with cardiovascular diseases (CVDs) as some of the leading contributors to global illness and premature mortality, as individuals suffering from such mental disorders often face an elevated risk of experiencing suicidal thoughts and encountering various other health complications [245,246,247]. As only a minority of patients experience complete remission following their initial course of treatment, we need to think beyond the already thoroughly studied mechanisms of these illnesses when discussing the potential pathogenesis [248]. This is where the immune system comes into play. In the past, diseases of the nervous system and the immune system were thought to be two separate entities, with interactions mainly linked to brain conditions such as multiple sclerosis. Recent studies have, however, shown a more complex and dynamic relationship, showing that the brain exhibits high levels of immunological activity and sophisticated innate immune responses and that it is not immunologically isolated from the periphery but rather communicates continuously with it [1].

Immune cells derived from bone marrow typically have limited access to the CNS due to the CSF and BBB. However, if these barriers become impaired by events such as enhanced metalloprotease activity, tight junction protein loss, endothelial cell degeneration, or in case of infection, increased BBB permeability, peripherally produced cytokines and immune cells no longer face any obstacles and can freely infiltrate the CNS, resulting in neuroinflammation and brain function abnormalities [249,250,251]. It is important to note that the CNS can produce cytokines internally by its innate immune cells, rather than only receiving them from the periphery. These immune cells, known as microglia, display different structural characteristics based on whether they are in an activated or resting state. When microglia are tasked with surveilling the central nervous system for possible dangers, they take on a morphology that allows them to coexist harmoniously with nearby neurons, astrocytes, and oligodendrocytes. Microglia, on the other hand, adopt an amoeboid morphology when a neuroinflammatory environment is present, and they release pro-inflammatory cytokines like interleukin 1β, interleukin 6, tumor necrosis factor α, interferon γ, chemokines like CCL2, and neurotransmitters like glutamate, adenosine triphosphate (ATP), nitric oxide (NO), reactive oxygen species (ROS), and reactive nitrogen species (RNS). It is noteworthy to mention that such changes in microglial morphology can also be triggered in response to tissue injury, stress, and infections, and can result in the disruption of neuro-glial processes important for maintaining balance inside the CNS [1,252,253].

**Figure 4:**
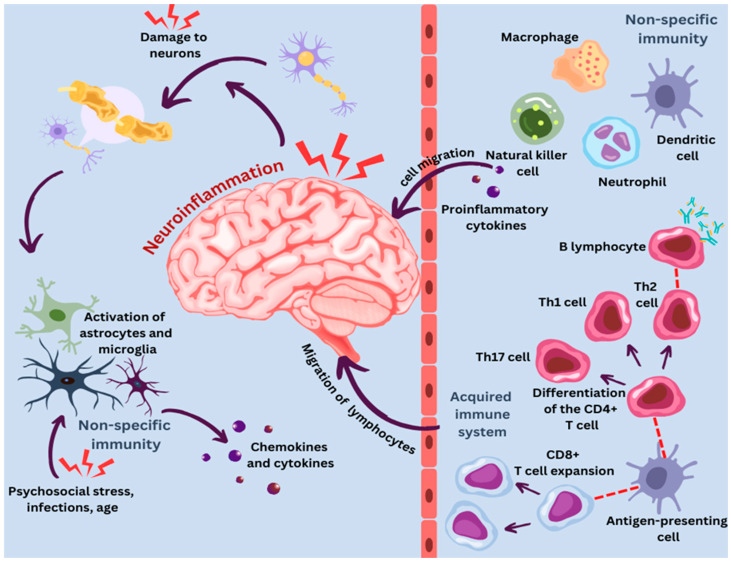
Neuroimmunomodulation’s involvement in mood disorders hinges on complex interactions between the nervous and immune systems. Notably, microglial activation emerges as a crucial factor. These resident immune cells respond to stress by releasing pro-inflammatory cytokines, contributing to neuroinflammation. Stress, a potent trigger, induces glucocorticoid release, further activating microglia and intensifying neuroinflammatory responses. Additionally, peripheral cytokines, produced by the innate and adaptive immune cells, infiltrate the brain through the disrupted blood–brain barrier, disturbing neurotransmitter balance and neuronal function.

While discussing stress as a potential trigger for these events, the precise activation mechanism of the innate immune response remains unknown. Nevertheless, research has demonstrated that a range of signals, including catecholamines, glucocorticoids, gut microbiota, and tissue alarm signals, may significantly contribute to this phenomenon referred to as “sterile inflammation” [254]. According to recent studies, the activation of neuroinflammation specifically induced by stress is thought to be one of the most common initiating factors in the pathophysiology of mood disorders, particularly depression [255]. Last but not least, neuroinflammation within the brain is characterized not only by microglial activation and high levels of pro-inflammatory cytokines, as already mentioned above but also by peripheral leukocyte infiltration and nerve tissue injury [256]. In light of the presented information, it is evident that normal brain function depends on a balanced neuroimmune system, and any disturbance or dysregulation within this intricate system can set in motion a series of pathological processes, ultimately culminating in the development of mood disorders (Figure 4).

### 5.1. Decoding Neuroinflammation in Depression

MDD, commonly known as depression, is a severe psychiatric condition with an estimated prevalence of 264 million globally. It is characterized by persistent symptoms such as sadness, altered psychomotor activity, and cognition, low energy, disrupted sleep and appetite, and poses a significant burden on daily life and psychosocial functioning [257,258,259]. Despite many interventions, there has been no reduction in the global prevalence or burden of depressive disorders since 1990, emphasizing the persistent and substantial impact of these conditions [260]. Research indicates that the rate of achieving remission following the initial treatment phase remains below 40%, despite the existence of several therapeutic choices for addressing MDD [261]. However, recent research has demonstrated that major depressive disorder might be associated with the immune system’s activation of its inflammatory response, suggesting that new therapeutic options may become available shortly [262]. According to the neuroinflammation theory, immune system imbalances brought on by stress amplify the central nervous system’s inflammatory response. This can be observed in MDD as long-term stress increases the permeability of the BBB and triggers the release of pro-inflammatory cytokines, which are a major contributor to depression.

A great influence on depression’s chronic inflammation is the increased activity of the sympathetic nervous system, which enables certain immune cells to enter the brain and activate microglial cells. These cells in turn produce pro-inflammatory cytokines like IL-1, IL-2, IL-6, IL-18, TNFα, and IFNγ, which are accompanied by a decrease in the levels of anti-inflammatory cytokines such as IL-4 and IL-10. Most important findings are summarized in Table 1. Elevated pro-inflammatory cytokine levels in individuals with depression may also prompt the release of cortisol via the hypothalamic–pituitary–adrenal (HPA) axis, leading to a loop of impaired inflammatory regulation and suggesting their potential role as biomarkers for depression [263,264,265,266]. Neuroinflammatory processes have been implicated in influencing specific brain regions, contributing to the pathogenesis of depression. These include the reward circuit, comprising the anterior cingulate cortices, ventral tegmental area, ventral striatum, ventral pallidum, raphe nucleus, and orbital prefrontal cortex, as well as the lateral habenula (LHb), known as the aversive center [266,267]. In suicidal patients with depression, autopsies revealed elevated primed microglia density in the dorsal anterior cingulate cortex (ACC), leading to persistent neuroinflammation affecting neuronal function. PET studies demonstrated increased binding of the translocator protein, indicative of increased microglial activation, in the ACC, hippocampus, insula, prefrontal cortex, and temporal cortex. This elevation in binding strongly correlated with the severity of depression [267,268].

Patients with significant depression have impaired connectivity in the ventral striatum, a critical portion of the brain’s reward system, especially when it comes to the degree of inflammation as shown by CRP levels. This altered connectivity extends to various brain regions, affecting networks associated with emotional regulation and reward processing, leading to depressive symptoms. Recent research demonstrates that during the anticipation of small rewards, there is a specific reduction in ventral striatal activation in those with elevated inflammation levels [267,269]. The dorsal raphe nuclei (DRN), a key hub of serotonin neurons in the brainstem, are closely linked to psychiatric disorders like anhedonia, anxiety, and depression. In response to inflammatory stimuli, the DRN experience microglial activation, changed gene expression, and neuronal alterations. These results suggest that DRN inflammation plays a significant role in the development and progression of depressive-like behaviors associated with conditions like inflammatory bowel diseases [267,270,271]. The LHb, implicated in various animal models of depression, consistently exhibits increased activity and is associated with psychiatric disorders, particularly major depression. Increased activity and βCaMKII expression in LHb were found to impact the serotonin (5-HT) neuronal activity in the DRN, providing a potential neurobiological link through which LHb contributes to the development of depression-like behaviors in illnesses associated with chronic pain [267,272].

**Table 1:** Comparative analysis of biomarkers in studies concerning depression.

Authors	Biomarkers Studied	Conclusions
Charlton et al. (2017) [273]	IL-1β, IL-6, TNF-α	IL-1β and IL-6 levels are elevated in the Late-Life Depression (LLD) group compared to healthy controls, with statistically significant differences. The TNF-α level is also higher in the LLD group, but the difference is not statistically significant at the given alpha level.
Wang et al. (2019) [274]	IL-1β, IL-6, TNF-α, IFN-α2, IFN-γ	IL-6 shows a significant elevation in patients with MDD compared to healthy controls. IL-1β, TNF-α, IFN-α2, and IFN-γ did not exhibit significant differences between the two groups.
Vogelzangs et al. (2016) [275]	CRP, IL-6, TNF-α, IFN-γ, IL-2, IL-4, IL-8, IL-10, IL-18, MCP-1, MIP-1α, MIP-1β, MMP2, TNF-β	In individuals with current depressive or anxiety disorders compared to healthy controls, elevated levels were observed in CRP, IL-6, IL-8, IL-18, MCP-1, MIP-1α, MIP-1β, MMP2, and TNF-β, while TNF-α, IL-10, IFN-γ, IL-2, and IL-4 levels either showed no significant elevation or were lower.
Dahl et al. (2014) [276]	IL-1β, IL-1Ra, IL-5, IL-6, IL-7, IL-8, IL-10, G-CSF, IFN- γ, MIP-1α, TNF-α, IL-2, IL-15	Plasma concentrations of IL-1β, IL-1Ra, IL-5, IL-6, IL-7, IL-8, IL-10, G-CSF, IFN- γ, and TNF-α are significantly elevated in patients with MDD compared to healthy controls. IL-2, IL-15, and MIP-1α did not show significant differences in plasma concentrations between the two groups.
Schmidt et al. (2014) [277]	IL-2, IL-4, IL-5, IL-10, IL-12, IL-13,GM-CSF, IFN- γ, TNF- α	In the depressed group compared to the non-depressed group, IL-5, IL-12, IL-13, GM-CSF, INF-g, and TNF-a levels were significantly elevated, while IL-2 and IL-10 levels showed no significant differences.

One of the pro-inflammatory cytokines that have sparked considerable research attention is TNF-α, which exhibited a noteworthy elevation in individuals diagnosed with MDD across numerous studies [278,279,280]. Its pivotal contribution to the development of depression lies in the escalation of the release of corticotropin-releasing hormone, adrenocorticotropic hormone, and cortisol, all of which hold significant functions within the hypothalamic–pituitary–adrenal (HPA) axis [281]. Another significant role of TNF-α lies in its induction of indoleamine 2,3-dioxygenase (IDO) activation, leading to the depletion of tryptophan, an essential precursor for serotonin. Due to IDO activation, this impact causes an increase in serotonin and tryptophan consumption, which provides a plausible explanation for the decreased availability of serotonin in depression [282]. Furthermore, TNF-α is associated with elevated plasma CRP concentrations both on the periphery and inside the central nervous system, which has been correlated with an elevated risk for a range of diseases, including cardiovascular disease, metabolic disorders, and diabetes, all of which are recognized as contributors to the development of MDD [283]. In a comparative analysis of patients with different rates of depressive episode onset, distinct patterns in serum cytokine levels emerged. Notably, individuals with rapid-onset depressive episodes, in contrast to those with a more gradual onset, exhibited reduced levels of TNF-α, along with other cytokines such as IFN-γ, IL-2, IL-4, IL-6, IL-10, and IL-10. Moreover, individuals with depressive episodes lasting fewer than six months displayed diminished serum levels of specific cytokines (IL-2, IL-8, IL-10, and IFN-γ) compared to those with episodes spanning 6 to 24 months [284].

In the context of managing treatment-resistant depression in individuals characterized by elevated baseline inflammatory markers, Infliximab, an anti-TNF-α antibody primarily indicated for autoimmune inflammatory conditions, has demonstrated the capacity to alleviate depressive symptoms [285]. When studied in individuals diagnosed with Crohn’s disease and ankylosing spondylitis, its effects on depressive symptoms have demonstrated promising results [286]. Additionally, a newly published meta-analysis examining Infliximab’s antidepressant effectiveness found that patients with elevated levels of inflammatory markers such as TNF-α and C-reactive protein benefited most from the medication [287]. These findings found further support in a study that examined Infliximab’s potential in alleviating treatment-resistant depression within a participant cohort of 60 people, exhibiting significant therapeutic benefits, particularly in those with heightened inflammatory markers [288]. Etanercept, on the other hand, falls within the same biological TNF inhibitor category but operates as a recombinant fusion protein of human TNF receptors. It competitively impedes the binding of endogenous TNF to cell-surface receptors, ultimately attenuating TNF’s effects [289]. Notably, it is generally acknowledged as a milder antagonist of TNF-α when compared to Infliximab [286]. Findings from a study indicate that Etanercept demonstrates effectiveness in alleviating anxiety and depression in psoriasis patients. However, it is noteworthy that sustained depression is linked to a reduced therapeutic response to etanercept [290]. Consistent with these results, an experimental study in mice has demonstrated that the extended administration of etanercept effectively reduces anxiety and depressive traits in diabetic mice [291]. Similarly, in a rat model of absence epilepsy, etanercept exhibited therapeutic potential in treating depression-like behavior [292].

As we expand our perspective to consider other innovative therapies, it is important to also highlight the potential of Pentoxifylline and Adalimumab as new and promising treatments for individuals with MDD. While both drugs have anti-inflammatory properties, the human immunoglobulin Adalimumab works by stopping TNF alpha from binding to specific receptors [293]. On the other hand, Pentoxifylline inhibits inflammatory reactions to pro-inflammatory cytokines like TNF by lowering their concentrations through an increase in cyclic adenosine monophosphate levels [294,295]. This was further demonstrated in a six-week, double-blinded, placebo-controlled trial involving 56 patients with MDD. When compared to a group receiving sertraline and a placebo, the combination treatment of pentoxifylline and sertraline significantly reduced depressed symptoms in patients [296]. In a similar study, the combination of adalimumab and sertraline dramatically improved depression symptoms and decreased inflammatory markers in a 6-week trial involving 36 patients with MDD. However, larger and longer-term studies are required to confirm these promising results in MDD treatment [297].

While many studies have reported increased levels of TNF-α in individuals with depression, it is essential to acknowledge that not all studies have consistently produced these positive findings [298]. This heterogeneity underscores the need for further research and a comprehensive understanding of the complex interplay between TNF-α and depression.

Similarly, IL-6 functions as a pro-inflammatory cytokine but differs from TNF-α. It is particularly important for the immune system, as it can boost the activity of B and T lymphocytes, trigger the acute phase response in response to infections and inflammatory processes, influence hematopoiesis, inhibit the growth of leukemic cells, and have additional effects on the nervous system [299,300]. High levels of IL-6 are mainly produced in adipocytes, highlighting a strong connection between dietary factors, obesity, and a higher incidence of behavioral problems—specifically, MDD and cognitive impairments—which are more common in obese individuals than in the general population [265,301]. However, it is important to recognize that both microglia and peripheral immune cells also contribute significantly to IL-6 production [265]. Moreover, IL-6 has been linked to brain signaling linked to “sickness behavior”, a compensatory response to illness or injury that manifests as behavioral changes like decreased activity and appetite as well as social changes like heightened feelings of social disconnection, loneliness, and sensitivity [302]. Apart from the observed decrease in neurogenesis linked to IL-6 signaling in the hippocampus, which is in line with the smaller hippocampus frequently observed in people with depression diagnoses, it was also shown that there was a significant reduction in prefrontal cortex thickness in association with elevated serum levels of IL-6 [303,304].

When considering potential mechanisms behind the pathogenesis of depression, it is worth mentioning that IL-6 has an inhibitory effect on the serotonin transporter (SERT), which plays a crucial role in regulating serotonin levels in the CNS. This inhibitory function was evidenced by a decrease in SERT activity, as well as reduced SERT protein and mRNA levels in both mouse hippocampal tissue and human choriocarcinoma JAR cells. Yet, it was shown that this effect was reversed in mice deficient in IL-6, whose hippocampal SERT levels were higher and whose depression symptoms were lower [305]. A six-year longitudinal study that looked into the connection between IL-6 and MDD discovered a similar cross-sectional relationship regarding IL-6 levels and an existing depressive disorder. Furthermore, the research discovered that among women with a baseline diagnosis, increased IL-6 levels over time were linked to a chronic course of depression [306]. Another recent study explored the regulatory role of IL-6 in depression-like symptoms using two rat depression models: chronic unpredictable mild stress (CUMS) and lipopolysaccharide (LPS) administration-induced depression. In the CUMS model, rats displayed a core depressive symptom known as anhedonia, along with behavioral despair in the forced swim test. IL-6 expression in the Cornu Ammonis 1 (CA1) hippocampus region was examined, revealing an initial significant increase in IL-6 mRNA levels during the first two weeks of CUMS exposure. However, both the prolonged CUMS exposure and the LPS-induced depression model led to a significant reduction in IL-6 mRNA levels [307].

In terms of treatment, utilizing IL-6 receptor antibodies or IL-6 antibodies to reduce depressive symptoms represents a new therapeutic strategy. For instance, sirukumab, a human anti-IL-6 monoclonal antibody, effectively blocks IL-6-mediated signaling and its biological effects through its high-affinity binding to IL-6 [308]. In those suffering from rheumatoid arthritis, a condition where depression-related symptoms are linked to high IL-6 levels, treatment with sirukumab significantly improved the reduction of depressive symptoms by the eighth week [309]. Another study examined the effectiveness of sirukumab in 36 patients with Cutaneous Lupus Erythematosus (CLE) or Systemic Lupus Erythematosus (SLE). The results indicated that sirukumab significantly improved mental health outcomes, particularly in CLE patients, highlighting its potential for enhancing well-being and mood in individuals with immune-mediated diseases [310].

Another anti-IL-6 antibody worth mentioning when talking about treating depression is tocilizumab, whose function is to inhibit the activation of both membrane-bound and trans-receptor signaling, as described in two published studies that confirmed its positive effect on symptoms related to MDD [311]. The significance of tocilizumab when treating depression and anxiety in rheumatoid arthritis patients was demonstrated in a study involving 91 adult patients with RA who received tocilizumab injections for 24 weeks, with approximately 66% of patients experiencing reduced anxiety and/or depression during the study [312]. In view of the COVID-19 pandemic, a study investigated the efficacy of tocilizumab as a therapy for intermediate to severe COVID-19 pneumonia, with an emphasis on depression, anxiety, and quality of life. Results revealed that patients in the tocilizumab group initially reported higher levels of depression, anxiety, and reduced quality of life at three months compared to the control group; however, the psychological well-being and quality of life improved for both groups at the six-month follow-up [313]. Unfavorable side effects from tocilizumab therapy mainly revolve around metabolic processes, including significant weight gain and increased cholesterol and triglyceride levels [301].

Contrary to conventional antidepressant drugs, ketamine is a noncompetitive N-methyl-D-aspartate (NMDA) glutamate receptor antagonist that was first licensed for use as an anesthetic. Over the past two decades, ketamine has gained attention for its potent antidepressant effects, especially in patients with treatment-resistant MDD [314,315]. To support this assertion, a 2020 study measured the inflammatory cytokine levels of 60 patients with MDD after they received six ketamine infusions. The results showed that the administration of ketamine was associated with decreased levels of IL-6 and other pro-inflammatory cytokines, linking this improvement to a reduction in depressive symptoms [316]. Intranasal ketamine has shown similar effectiveness in treating MDD, delivering improvement with only mild and temporary adverse effects [317]. Given that ketamine lacks FDA approval for treating depression, it is utilized as an off-label intervention with limited exploration into its long-term benefits. This highlights the necessity for further randomized controlled trials to establish the efficacy and safety of all forms of ketamine in the treatment of depression.

### 5.2. Understanding the Role of IL-33 in Depression: Insights from Meta-Analysis and Biological Mechanisms

IL-33, a pro-inflammatory cytokine from the IL-1 family, is expressed in numerous types of cells, such as microglia and astrocytes [318]. These cells show a high expression of its receptor, ST2 [319]. IL-33 has a critical role in brain areas important for emotional function, implying its importance in depression development [320]. Upon inflammatory stimulation, cells produce IL-33, which activates downstream pathways that regulate pro-inflammatory and Th2-related cytokines, making it an important participant in the cytokine hypothesis of depression [321].

Earlier research reveals that IL-33 has a dual role as a pro-inflammatory factor influencing depression development and a neurotrophic factor controlling depression development [322]. Despite numerous studies on IL-33 and depression, encompassing variations in circulating levels throughout the illness and electroconvulsive treatment, the findings are conflicting [323,324,325]. To address this, a meta-analysis was carried out to determine IL-33’s particular influence on depression, providing a new viewpoint on immunological depression therapy.

IL-33 influences central nervous system synapses by regulating microglial phagocytosis, notably in regions related to emotions such as the thalamus [326]. As a member of the IL-1 cytokine family, IL-33 has two functions: as a transcriptionally inhibitory N-terminal domain (aa1-78) and as a pro-inflammatory IL-1-like cytokine domain (aa111-270) that interacts with ST2L [327]. Early neurodevelopmental cleavage of proIL-33, which is generated by neural glial cells, influences pro-inflammatory processes in the brain via the IL-33/ST2/AKT pathway. This influence affects mitochondrial activity, microglial polarization, and synaptic remodeling, which may help prevent depression and neurodegenerative diseases [322,328,329].

Moreover, IL-33 regulates microglial activation and polarization, potentially affecting anxiety control in the basolateral amygdala via the IL-33/ST2/NF-κB pathway [330]. However, contradictory data show that IL-33 may suppress brain-derived neurotrophic factor (BDNF) synthesis via the NF-κB pathway [328].

In terms of its function in depression, IL-33 mRNA levels are greatest in the brain and spinal cord, particularly in stress-responsive areas such as the paraventricular nucleus and prefrontal cortex [331,332]. Stress and inflammation activate IL-33 expression, which influences midbrain nucleus biogenic amine metabolism, HPA axis activity, cortisol levels, and neurotrophic factor downregulation [333].

IL-33’s effect on synaptic remodeling in microglia and astrocytes affects emotion-related brain areas, potentially influencing depression risk [334]. While direct research on chronic stress and depression is scarce, a theoretical theory proposes an indirect link between IL-33, human microglia, and depression triggers, which influences neurodevelopment and synapse count. Additional study is required to confirm these findings.

Due to its magnitude and unclear cytokine balance with serum, IL-33 in cerebrospinal fluid presents difficulties that make it challenging to consistently determine its impact on depression [335]. By controlling the development of the central nervous system, microglial cells produce IL-33 in the brain, which affects memory and emotion-related areas, synaptic remodeling, and depression [336]. In patients with bipolar illness, major depressive disorder during pregnancy, postpartum depression, and Alzheimer’s disease, there are elevated levels of IL-33 in the cerebrospinal fluid [337,338,339]. Although the link between dose and response is yet unknown, IL-33 may cause tryptophan breakdown and consequent depression [340].

Despite its high concentration, serum IL-33 exhibits correlations with depression, which may be explained by active transport and blood–brain barrier leakage [335]. Central and peripheral IL-33 levels may be regulated by the HPA axis [341]. Serum IL-33 has a clear correlation to depression among female patients who have experienced abuse as children in the past, alopecia areata, systemic lupus erythematosus, and recurrent major depressive disorder [342,343,344].

Although there is inconsistent research on IL-33’s effects on depression, research points to a protective effect. Increased levels of circulating IL-33 are linked to a decreased incidence of depression, and IL-33 corresponds with a lower recurrence rate in MDD and BD electroconvulsive treatment [345]. Particularly among women with a history of childhood maltreatment, some single-nucleotide polymorphism (SNP) haplotypes in the IL-33 gene, such as rs11792633 and rs7044343, offer protection against depression [344]. Depression is a result of neurodevelopmental degenerative alterations, and IL-33 influences synaptic quantity and remodeling by functioning as a neurotrophic factor and a pro-inflammatory factor [330].

Under normal circumstances, IL-33 signals are produced by maturing microglia and promote mitochondrial metabolism, M2-type macrophage polarization, and synaptic phagocytosis and remodeling. This suggests that IL-33 is essential for preserving the number of synapses and neurodevelopment in the thalamus and spinal cord at normal levels. Changes in circulating ST2 levels reveal a positive link with lower depression risk, consistent with IL-33 effects. ST2, the receptor for IL-33, is correlated with depression [346]. Nevertheless, no research has found a link between depression and ST2-related SNPs. The HPA axis, neuroinflammation, and monoamine signaling were supported by IL-33 participation in an experimental study on male mice that found that persistent stress produced anxiety or depression-like behavior. This finding offers fresh insight into the function of IL-33 in regulating the development of depression.

People with BD and MDD exhibit different immunological patterns in their immune-inflammatory response system (IRS) and compensatory immune regulatory response system (CIRS) [347]. In BD, IL-33 or ST2 mostly controls the IRS and CIRS, providing antidepressant protection. Subgroup analysis suggests that elevated IL-33 or ST2 levels are beneficial for both MDD and BD. The effects of IL-33 on depression among animals have been confirmed [348]. Antidepressant medication’s effect on circulating IL-33 or ST2 in some patients may lead to false negative correlations, even though several studies have found no significant relationship between the two variables and depression. ELISA is the most widely used technique for measuring cytokines, including IL-33, and yields reliable results on a variety of platforms. Subgroup studies demonstrate the preventive function of IL-33 against depression, independent of the cause or course of therapy. Potential factors influencing study heterogeneity are highlighted using meta-regression and sensitivity analyses. These parameters include significance, depression kinds, ethnicity, and genes. This raises the possibility of IL-33 as a depression diagnostic and treatment tool. IL-33 levels can be measured in depressed patients to help with prognosis, diagnosis, and treatment planning. The IL-33/ST2/NF-KB pathway and SNP haplotypes provide possibilities for focused treatment interventions, highlighting the significance of caution and care in patients with low IL-33 levels or certain genetic markers [349].

## 6. Bipolar Disorder: Immunological Insights

Bipolar disorder includes bipolar disorder type I (BD-I), bipolar disorder type II (BD-II), and cyclothymic disorder, which are chronic mental illnesses marked by frequent episodes of mania or hypomania interspersed with periods of depression. Behavioral characteristics of bipolar I disorder are mainly described as manic episodes, which can include delusions and hallucinations as much as 75% of the time. Manic episodes can also occasionally be accompanied by depressive symptoms. In contrast, bipolar II disorder is predominantly defined by episodes of depression alternating with hypomania without the occurrence of manic phases.

Regarding prevalence, bipolar I disorder is estimated to have a global lifetime occurrence rate ranging from 0.6% to 1.0%, while bipolar II disorder falls within a range of 0.4% to 1.1%. Finally, but just as importantly, cyclothymic disorder is defined as a mix of hypomanic and depressive symptoms with episodes that last for at least two years, but neither condition fully satisfies the diagnostic requirements for either bipolar disorder or major depressive disorder [350,351,352,353].

Despite the existence of a broad spectrum of treatments for bipolar disorder that are currently accessible, the current pharmacological options fall short of addressing the high rates of relapses and recurrences. Since BD lacks a singular common cause, its pathophysiology and etiology remain incompletely understood. However, recent studies point to immune system activation and elevated cytokine levels as factors in the development and progression of this condition in a significant subset of cases [354]. Two terms that are frequently used when discussing neuroinflammation as a pathophysiological mechanism for BD are the IRS and the CIRS. The CIRS serves as a reactive mechanism triggered by the IRS, playing a crucial role as a regulatory feedback system. The CIRS acts to counteract any inflammatory response that the IRS initiates by raising the amounts of anti-inflammatory cytokines [355,356]. The CIRS typically produces interleukin 4, interleukin 10, and transforming growth factor-α in response to elevated levels of interleukin 1, interleukin 6, tumor necrosis factor, interferon-γ, interleukin 2, and interleukin 17 [357].

Furthermore, a growing body of research has repeatedly shown that BD is linked to increased levels of pro-inflammatory cytokines, indicating a possible connection between aberrant immune responses and the onset or progression of the illness [354].

### 6.1. Exploring Correlations: BD-II Candidate Proteins, Cytokines, and BDNF

Potential protein biomarkers for BD-II, such as PRDX2, CA-1, FARSB, MMP9, and PCSK9 have also been discovered by certain researchers. These biomarkers work well together for diagnosing BD-II. It is yet unknown, though, how these proteins contribute to the pathophysiology of BD-II [358]. Elevated TNF-α, CRP, and IL-8 levels during acute episodes, along with decreased levels post-treatment, suggest cytokines as potential diagnostic and staging biomarkers [359]. Significant manic and depressed symptoms as well as later phases of bipolar disorder are correlated with brain-derived neurotrophic factor, which is essential for neuron formation and may serve as a biomarker for the status and progression of the condition [360,361,362].

Drawing from prior research indicating the inflammatory characteristics of specific proteins associated with BD-II, a recent study explored relationships between these BD-II candidate plasma proteins, cytokines, and BDNF [363,364,365]. The aim was to elucidate correlations between patients and controls. Anticipating potential associations and differences in correlations, the study provided new insights into underlying mechanisms.

In a separate study, significant connections were found between cytokine and BDNF levels and the plasma levels of potential BD-II proteins [358]. These links, however, differed between the control group and BD-II patients. Through their association with inflammatory markers, the study provided preliminary evidence about these potential proteins’ role in the mechanisms behind BD-II.

### 6.2. FARSB Protein and BDNF

According to recent studies, BD-II patients’ plasma Phenylalanyl-TRNA Synthetase Subunit Beta (FARSB) protein and BDNF levels are significantly higher than those of controls [358,361,366,367,368]. Furthermore, there was a clear positive association in both groups between the levels of BDNF and FARSB. Contradictory findings on BDNF levels in BD stages necessitate further exploration [368,369]. Moreover, another study suggested elevated BDNF levels in early-stage BD-II, potentially indicating a compensatory or protective effect, as observed in individuals with familial BD risk [366,368].

FARS2, an enzyme linked to aminoacyl-tRNA synthetase-related diseases, demonstrated correlations between both BDNF and IL-8 [363,364,365,370,371]. The positive correlation with IL-8 supports the role of inflammation in BD-II pathogenesis. Furthermore, it has been proposed that FARSB, acting as a neurotransmitter in response to neuronal injury, may trigger BDNF compensation [370,371].

### 6.3. CA-1 and IL-8 Levels

In postmortem tissue, changes in Carbonic Anhydrase 1 (CAR1) were seen in the frontal brain. A zinc-metalloenzyme called CAR1 makes it easier for carbon dioxide (CO_2_) to be reversibly hydrated. Carbon dioxide and water are converted into protons (H^+^) and bicarbonate ions (HCO_3_^−^) upon the activation of CAR1. An alkaline extracellular environment is produced by decreased CAR1 levels, which also result in reduced extracellular bicarbonate ions and protons.

CAR1 is an essential regulator of neuronal excitability and synaptic transmission, as it governs the release of protons (H^+^) and bicarbonate into the extracellular gap. Synaptic transmission is directly impacted by protons (H^+^). Through its particular modulation of pH and the bicarbonate concentration in the hilus area of the hippocampus, CAR1 influences inhibitory neuronal transmission, which in turn affects granule cell (GC) excitability. Restoring CAR1 expression in the astrocytes of mice lacking CAR1 compensates for impairments in granule cell inhibitory neuronal transmission. The ventral hippocampus of mice exposed to pharmacological stimulation or overexpression of CAR1 exhibits notable impacts on synaptic transmission and neuronal activity. These results demonstrate the critical function of CAR1 in brain functions and shed light on its functional importance outside of depression [372].

BD-II patients had significant increases in their levels of CA-1 and IL-8, and only in the BD-II group was there a positive association between the two. The primary component of many cells, CA-1 catalyzes the conversion of carbon dioxide and water. The association with IL-8 supports recent studies that indicate CA-1 participation in BD-II pathophysiology through the cytokine system [369,373,374].

In addition, CA-1 exhibited significant correlations with TNF-α, and Matrix metalloproteinase-9 (MMP9) correlated with CRP, specifically in the control group [375,376,377,378]. MMP9’s role in increasing BBB permeability and its association with inflammatory cytokines align with its positive correlation with CRP in normal controls [377]. Whether MMP9 and CRP can serve as biomarkers for other inflammatory diseases requires further investigation [375,376]. These findings shed light on the intricate relationships between candidate proteins, cytokines, and BDNF in BD-II, contributing to a deeper understanding of the disorder’s underlying mechanisms [358].

### 6.4. Calcium Signaling and ER Stress in Bipolar Disorder: Insights and Mechanisms

In addition to the malfunction of the mitochondria and oxidative system, changes in calcium signaling and stress responses associated with the endoplasmic reticulum (ER) are frequently seen in BD in post-mortem, clinical, cellular, and imaging studies [379,380]. Calcium, akin to ROS, serves as a potent activator of NLRP3 [381]. Calcium ions play vital roles in modulating neuronal functions. Even slight changes in the minute fraction (<1%) of free intracellular calcium can significantly impact neuronal function and trigger apoptotic cascades [379,380,382]. Patients with BD can be diagnosed by common single-nucleotide polymorphisms (SNPs) in voltage-gated calcium channel genes, specifically at the CACNA1C locus [380,382,383]. Human neurons that have been induced from high-risk BD genotypes show improved calcium signaling and higher expression of the CACNA1C gene. It is noteworthy that in samples from patients who respond clinically to lithium, the drug specifically reverses this hyperexcitable phenotype [384,385,386,387]. Through mitochondria-associated membranes (MAMs), endoplasmic reticula and mitochondria work together to control the intracellular calcium balance. MAMs function as locations where NLRP3 complexes assemble and serve as sensors for elevated ROS generation from damaged mitochondria, which triggers the release of cytokines that promote inflammation [381].

### 6.5. Kynurenine Pathway

In BD, tryptophan metabolism via the kynurenine (KYN) pathway plays a critical role in mediating the relationship between cellular stress and systemic inflammation, impacting a range of physiological processes [388,389]. TRP is converted to KYN by stress hormone-regulated enzymes called extra-hepatic indoleamine 2,3-dioxygenase (IDO) and intra-hepatic tryptophan dioxygenase (TDO). Patients with euthymic bipolar disorder show a higher conversion of TRP to KYN, with more pronounced effects in the central nervous system [390]. Astrocytic metabolism produces neuroprotective kynurenic acid, while microglial processing leads to neurotoxic quinolinic acid and 3-hydroxykynurenine (3HK) [389].

Inflammatory conditions enhance kynurenic toxicity, disrupting the blood–brain barrier and elevating pro-inflammatory cytokines [388]. Reduced hippocampus functioning and depressive symptoms are linked to imbalances in KynA/QA ratios in a number of illnesses [389]. By aggravating the conversion of TRP to KYN, metabolic inflammation connects kynurenine signaling to BD symptoms [390]. Preclinical studies support this link, revealing protective effects against obesity-induced inflammation in IDO-knockout mice [389].

**Figure 5:**
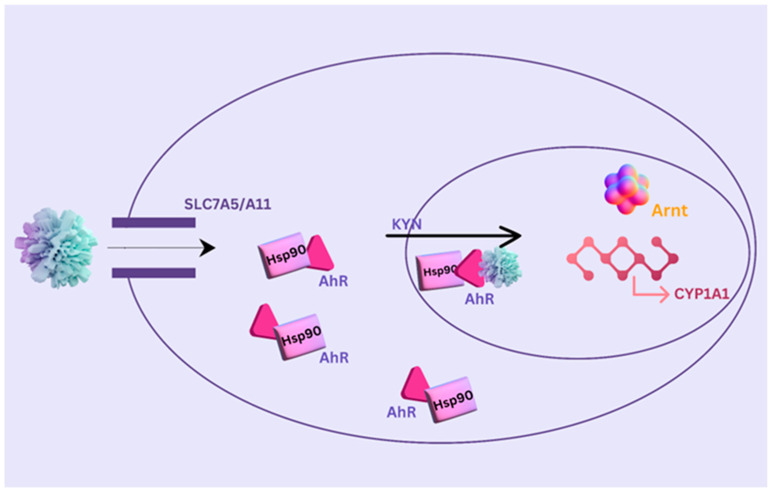
The consistent interaction of the aryl hydrocarbon receptor (AhR) with heat-shock protein (Hsp90) in the cytoplasm of the cell is a necessary component of kynurenine (Kyn) signaling via AhR. Through the transporters SLC7A5 and SLC7A11, Kyn enters the cell and attaches itself to the Hsp90-AhR complex. Following that, this coupled complex travels to the nucleus, where AhR binds to the aryl hydrocarbon receptor nuclear translocator (Arnt) protein to activate targets farther along the chain, such as Cyp1a1.

Interestingly, blocking IDO can prevent the depression brought on by LPS, and low-dose ketamine acts as an antidepressant by influencing KynA/QA competition at the NMDA receptor [391]. Activated by KYN, the aryl hydrocarbon receptor (AhR) impacts mitochondrial activity and plays a role in inflammatory reactions [392,393]. By causing neuroprotective changes in kynurenine metabolites, therapies including electroconvulsive therapy (ECT), exercise, and cyclo-oxygenase (COX) inhibitors are effective in treating BD and MDD [389]. Key molecules known to date and their interactions are presented in Figure 5.

Clinical trials investigating KynA analogs and IDO inhibitors are underway, but caution is advised due to potential effects on cognitive and psychotic symptoms [394]. Importantly, physicians need to exercise caution when treating patients with cognitive and psychotic symptoms since KYN modulation may have unexpected effects [389,395,396].

### 6.6. NLRP3 Inflammasome: Linking ER/Mitochondrial Stress to Immune Activation in Bipolar Disorder

Periods of heightened ER/mitochondrial stress can lead to substantial protein/calcium imbalances, initiating apoptotic and neuroinflammatory responses. The NLRP3 inflammasome, comprising NLRP3, ASC, and caspase-1, plays a pivotal role in this process. Upon induction, NLRP3 colocalizes with MAMs, contributing to mitochondrial destabilization and further activation of NLRP3. The two-step activation involves Toll-like receptor priming and subsequent activation through various effectors like UPR, ROS, mtDNA, Ca^2+^, lipids, purines, and pathogens [397].

When these signals are recognized, caspase-1 activates, causing pyropoptosis and the production of pro-inflammatory cytokines (IL-1β and IL-18) [398]. The inflammatory cycle, triggered by cell death and cytokine release, can lead either to damage repair or chronic disease progression. This notion is supported by a post-mortem study of frontal cortex samples from patients with BD, which shows reduced levels of mitochondrial complex I and higher levels of NLRP3, ASC, caspase-1, and cytokines [399].

NLRP3’s role extends to metabolic disorders, and medicines that interact with NLRP3 have demonstrated a benefit in treating some BD symptoms. The relationship between insulin resistance and peripheral IL-1β levels in bipolar patients and suicide risk highlights the importance of the inflammasome in immunological response and cardiometabolic illness. Medications exacerbating metabolic syndrome in BD raise concerns, urging further exploration of NLRP3 activity in common treatments.

Potential therapies focusing on NLRP3 inhibition, such as Baicalin and ketogenic diets, show promise in preclinical studies [400]. Baicalin exhibits antidepressant effects and mitigates obesity and insulin resistance. Ketogenic diets, known for their benefits in epilepsy, also demonstrate positive effects on mood and inflammation, possibly through NLRP3 inhibition [401]. Trials have begun to explore the safety and effectiveness of these therapies, indicating a possible role as adjuvant therapy in BD, particularly for individuals with elevated metabolic burden [359].

## 7. Unraveling Schizophrenia: Pathogenesis and Immune Links

Schizophrenia is a severe psychiatric disorder identified by clinical features that include positive and negative symptoms, mood alterations, disorganization symptoms, and cognitive impairments. This condition affects nearly 1% of the global population, presenting a significant mental health challenge [402,403]. Its onset typically occurs in the late teens or early twenties, often preceded by a prodromal phase marked by subtle behavioral changes. The heritability of schizophrenia is considerable, with genetic factors accounting for 80% of the risk, and studies implicate immune system pathways and synaptic function. The immune hypothesis in schizophrenia suggests a genetic disturbance that increases vulnerability to psychosis, evident in genetic studies indicating overlap between schizophrenia-associated genes and those related to immune function.

During the prodromal phase, individuals with schizophrenia exhibit anomalies such as reduced gray matter volumes and dendritic irregularities, especially in the prefrontal and parahippocampal regions. Signs of neuroinflammation are suggested by the association between the reduction in gray matter and increased immune markers, such as tumor necrosis factor α. This implies the possibility of the cytokine-mediated activation of microglia in this disorder, with elevated cytokine levels showing a correlation with the severity of symptoms. These structural aberrations contribute to the modification of physiological activity and the alteration of functional connectivity within critical brain regions associated with schizophrenia [404,405].

### The Immune System’s Role in Schizophrenia: Exploring the Connection

Similar to previously mentioned mood disorders, the pro-inflammatory cytokine IL-6 plays a significant role in the neuroinflammatory pathogenesis of schizophrenia, serving as a focal point in numerous studies. According to research, high levels of IL-6 are associated with negative effects on both hippocampal gray matter volume and white matter integrity in the brain. Higher levels of peripheral IL-6 have also been linked to detrimental effects on memory, learning, and general cognitive function [406].

These effects are exhibited by patients who struggle to shift attention between different tasks or activities, experience a negative influence on the ability to focus on visual stimuli, and have slower visual and motor information processing speeds [407]. According to several studies, certain cytokines may function as disease-specific markers of inflammation. This theory is supported by research showing that both relapse patients and those going through their first psychotic episode had higher IL-6 levels during the illness’s acute phase [408].

A newly published meta-analysis involving first-episode psychosis (FEP) patients revealed that IL-6 concentrations were noticeably higher in FEP individuals in contrast to healthy controls. Subgroup analyses confirmed the reliability of these connections, highlighting that IL-6 plays a crucial role in the biological processes associated with the development of first-episode psychosis [409]. Notably, there was a correlation between the total severity of psychopathology and IL-6 levels. Furthermore, psychotic symptoms in early adulthood were linked to elevated IL-6 levels measured in childhood, years before psychosis onset.

A comprehensive study involving 311 participants investigated this idea and revealed a correlation between elevated levels of the inflammatory marker IL-6 and occurrences of both childhood trauma and compromised social cognition [410]. In the context of examining neuroinflammation in chronic schizophrenia, a separate study unveiled elevated IL-6 levels in individuals with persistent schizophrenia, accompanied by heightened concentrations of various cytokines, including TNF-α, IL-12, INF-γ, and sIL2r [408]. Complementing these results, a different study examined cytokine fluctuations in serum during admission and discharge in individuals diagnosed with chronic schizophrenia. Clinical features upon discharge improved when IL-6 concentrations were lowered [411].

An extensive and recently updated review was conducted to assess the effectiveness of anti-inflammatory medications in the context of the evolving landscape of schizophrenia treatment. The review included 62 double-blind randomized clinical trials with 2914 patients. As the most researched COX-targeted anti-inflammatory drug, celecoxib, a specific COX-2 inhibitor, produced mixed results across four trials. Even though there was only one study that clearly demonstrated a distinction between celecoxib and a placebo, two other studies suggested that there might be an effect, and a third analysis corroborated the notion that adding celecoxib could significantly improve symptoms, particularly in those going through their first episode of schizophrenia.

Low-dose aspirin (a COX-1 inhibitor) demonstrated positive outcomes in two trials, especially in a subgroup with elevated baseline symptom severity, although concerns about potential side effects were raised. Overall, the effects of anti-inflammatory drugs such as celecoxib, aspirin, and minocycline on symptomatology and overall functioning were noteworthy. However, the exact mechanisms by which these drugs work so well in cases of schizophrenia are still unknown [412].

A link between inflammatory processes, led by higher values of CRP, is a common thread across multiple studies. A systematic review and meta-analysis of prospective cohort studies, involving 89 792 participants, looked into the connection between CRP levels and the subsequent development of psychotic disorders. The analysis unveiled a significant finding: individuals with elevated CRP levels at baseline exhibited a 50% higher risk of developing psychosis compared to those with lower levels. However, the strength of this association weakened upon excluding individuals with suspected infections and incorporating additional adjustments [413].

In both acute and chronic phases of schizophrenia, elevated levels of CRP have been observed, as indicated by a study exploring acute psychosis. This study demonstrated a relationship between positive symptoms in acute psychosis and CRP levels. On the other hand, different studies have explicitly found a connection with cognitive dysfunctions as opposed to positive symptoms. Furthermore, a meta-analysis involving five studies exploring cytokine levels in chronic schizophrenia patients disclosed that 28% of these patients manifested elevated CRP levels [408].

The role of CRP within the context of chronic inflammation has been identified as a significant contributor to reduced cognitive functions. This association was underscored by a study involving 208 patients with schizophrenia, where a decrease in CRP levels was found to be correlated with overall improvements in global cognitive performance. However, this association did not extend to individuals specifically within the schizophrenia spectrum. Interestingly, further cognitive alterations were linked to reduced CRP levels, emphasizing the complex interaction of inflammation, CRP, and cognitive function in schizophrenia [414].

A different study, however, offers an opposing perspective. It shows that while increased CRP levels are clearly associated with cognitive impairment in schizophrenia, the correlation’s comparatively small practical significance implies that inflammation may not have a significant effect on cognitive dysfunction in the majority of schizophrenia patients [415].

In exploring therapeutic avenues for schizophrenia, recent double-blind randomized placebo-controlled trials investigating the efficacy of adjunctive 1000 mg aspirin as an anti-inflammatory intervention presented unexpected outcomes. Contrary to expectations, neither Study 1, involving 200 patients, nor Study 2, with 160 patients exhibiting elevated CRP levels, revealed statistically significant differences in primary (overall symptoms in Study 1 and positive symptoms in Study 2) or secondary outcomes (other symptoms or patient well-being) between aspirin and a placebo. Furthermore, a meta-analysis that included previous research was unable to determine if supplementary aspirin medication significantly reduced symptoms of schizophrenia when compared to a placebo [416].

In contrast, there is increasing acknowledgment of the medicinal use of omega-3 polyunsaturated fatty acids, particularly docosahexaenoic acid (DHA) and eicosatetraenoic acid (EPA), in reducing inflammatory states. Numerous studies have linked increased consumption of these omega-3 fatty acids to a lower incidence of chronic diseases characterized by elevated inflammation [417]. Omega-3 fatty acid supplementation has been proven to significantly reduce TNF-α, IL-6, and CRP levels according to a comprehensive analysis of 68 randomized trials. This advantageous effect was noted in both healthy individuals and those with long-term autoimmune and non-autoimmune disorders, underscoring the comprehensive anti-inflammatory capabilities of omega-3 fatty acids [418].

Interestingly, approximately one-third of individuals with schizophrenia possess antigliadin antibodies of the immunoglobulin G type. This subgroup, characterized by elevated anti-gliadin antibody (AGA) IgG levels, is linked to a chronic inflammatory state caused by increased levels of peripheral cytokines passing through a leak in the BBB. Recognized as a distinct subset within the heterogeneous landscape of schizophrenia, individuals having high AGA IgG levels may indicate gluten sensitivity, leading to targeted interventions like a gluten-free diet. One such pilot study involving individuals with schizophrenia and elevated anti-gliadin antibodies showed that those following a gluten-free diet experienced significant improvements in their general psychiatric condition and negative symptoms in contrast to individuals following a gluten-containing diet [419]. These findings open a new avenue for alternative therapeutic approaches or precision treatments; however, further research is necessary to draw definitive conclusions and fully understand the implications of these discoveries.

## 8. Conclusions

From early 20th-century discoveries to the revolutionary discoveries of the 1970s and beyond, the evolution of neuroimmunomodulation has markedly advanced our understanding of the complex interaction between the immune and neurological systems. Important concepts of conditioned immunosuppression were established, providing the framework for this multidisciplinary area. Recent discoveries have clarified the roles of complementary systems, gut microbiota, and immunological senescence in neurodegenerative and neuroinflammatory disorders, emphasizing the complex relationship between aging and neurodegeneration and systemic and central nervous system inflammation, often referred to as “inflammaging”. In the evolving landscape of pain management and neurological therapies, there is a growing emphasis on developing innovative methodologies beyond traditional approaches like VNS and neuromodulation techniques such as TMS. These novel methodologies aim to target the complex neural circuits involved in pain perception, mood regulation, and cognitive functions, presenting new opportunities and challenges in clinical practice. While VNS and TMS have shown promise in modulating neural activity and providing therapeutic benefits for conditions like chronic pain, depression, and epilepsy, they also come with inherent limitations. VNS, for example, involves either invasive procedures or relies on patient compliance with non-invasive devices, while TMS requires specialized equipment and clinical settings, often limiting accessibility. Transcranial direct current stimulation, transcranial ultrasound stimulation, closed-loop neuromodulation, optogenetics and chemogenetics, and sophisticated imaging methods are examples of emerging technologies that have transformed diagnostic and therapeutic approaches and made more targeted interventions possible. While neuroimmune pharmacology continues to provide targeted treatments for neuroinflammatory conditions, other techniques, such as vagus nerve stimulation, neurofeedback, and biofeedback, have demonstrated encouraging results in modulating inflammatory responses, improving mental health, and enhancing cognitive functions. The investigation of neuroimmunodulation in the treatment of chronic pain highlights the potential benefits of glial inhibitors, neuromodulation methods, and anti-inflammatory drugs; nevertheless, there are still obstacles in the way of converting preclinical results into practical clinical treatments. The interaction between cytokines, like IL-33, and pathways, like the kynurenine pathway, has revealed new treatment targets in mood disorders, including major depressive disorder and bipolar disorder, and it has also shed light on the intricate role that neuroinflammation plays in mental health. Furthermore, the necessity of comprehensive methods that target the underlying mechanisms and symptoms of neurodegenerative illnesses and mood disorders is underscored by the role that systemic inflammation plays in aggravating these conditions. Clinical trials and ongoing interdisciplinary collaboration will be crucial to developing these medicines further, maximizing their use, and eventually enhancing patient outcomes as research advances. This multimodal approach highlights how important it is to comprehend and target the neuroimmune interface in order to improve our ability to effectively manage and cure neurological and inflammatory illnesses.

## Data Availability

No new data were created or analyzed in this study. Data sharing is not applicable to this article.
